# Association Between Vaginal Microbiota and Female Infertility: A Systematic Review and Meta-Analysis

**DOI:** 10.3390/microorganisms14092094

**Published:** 2026-09-18

**Authors:** Hanyue Zheng, Dengyan Qi, Mengjie Zhao, Yushu Wu, Jiechen Yin, Bei Wang, Xiang Hong

**Affiliations:** Key Laboratory of Environmental Medicine Engineering, Ministry of Education, School of Public Health, Southeast University, Nanjing 210009, China; 220244227@seu.edu.cn (H.Z.); 15548760957@163.com (D.Q.); zhaomj0819@163.com (M.Z.); 15906151529@163.com (Y.W.); jiechen_yin@seu.edu.cn (J.Y.); wangbeilxb@163.com (B.W.)

**Keywords:** infertility, vaginal microbiota, CST, meta-analysis

## Abstract

Vaginal dysbiosis may contribute to female infertility, but evidence on specific microbiota patterns remains inconsistent. This meta-analysis updates the epidemiological evidence linking vaginal dysbiosis to reduced female fertility, with a particular focus on low-*Lactobacillus* vaginal microbiota (LL-VMB), bacterial vaginosis (BV), intermediate microbiota, and Community State Type (CST) subtypes. A systematic search was performed in PubMed, Embase, Web of Science, Scopus, and the Cochrane Library from inception to 21 July 2025. We included observational studies that compared vaginal microbiota status with infertility outcomes in women of reproductive age. The primary exposure factors investigated were LL-VMB, BV, intermediate microbiota, and specific CST subtypes. Data were screened and extracted by two independent researchers. Thirty-one studies were included, with twenty-three eligible for meta-analysis. Evidence certainty was rated as very low. Compared with the control group, LL-VMB (OR = 2.16, 95% CI: 1.59–2.95) and BV (OR = 2.17, 95% CI: 1.54–3.06) were significantly associated with increased infertility risk. Intermediate microbiota showed no significant association. Exploratory network meta-analysis suggested potential variations in infertility risk among CST subtypes. Future large-scale, longitudinal, and multi-omics investigations must elucidate the mechanisms linking specific microbiota structures to reduced fertility.

## 1. Introduction

Infertility has emerged as a major global public health concern, imposing a significant burden on individuals, families, and healthcare systems [1,2]. The World Health Organization (WHO) estimates that approximately one in six adults will experience infertility in their lifetime, defining the condition as failure to achieve a clinical pregnancy after 12 months or more of regular, unprotected sexual intercourse [3,4]. The etiology of infertility is complex, with female factors accounting for roughly 40% to 50% of all cases. Common causes include ovulatory disorders, tubal factors, and endometriosis [5]. Despite current diagnostic protocols successfully identifying most underlying causes, approximately 15% to 30% of patients remain classified as having unexplained infertility following routine clinical evaluation [5].

As an essential component of the female reproductive system, the vaginal microbiota is closely related to fertility decline [6]. In healthy women of reproductive age, the vaginal microbiota is typically dominated by *Lactobacillus* species, most commonly *L. crispatus*, *L. iners*, *L. gasseri* and *L. jensenii* [7]. This *Lactobacillus*-dominant state maintains a low-pH environment that inhibits the colonization of opportunistic pathogens, thereby creating favorable conditions for conception [8,9]. Conversely, vaginal dysbiosis occurs when there is a depletion of *Lactobacilli*, an overgrowth of anaerobic bacteria, and an elevation in microbial diversity, classically presenting as bacterial vaginosis (BV) [10]. Extensive research has demonstrated an association between BV and a spectrum of adverse reproductive outcomes, including pelvic inflammatory disease (PID), preterm birth, and miscarriage [11,12]. Concurrently, growing epidemiological evidence suggests a strong association between BV and female infertility, which has been repeatedly observed in cases of tubal factor infertility and unexplained infertility [13,14,15]. However, vaginal dysbiosis is not limited to populations meeting the clinical diagnostic criteria for BV. Intermediate microbiota states are common in the general population, and prior evidence indicates they may also elevate the risk of infertility [16]. Consequently, describing exposure solely through a binary “BV versus non-BV” classification may overlook the potential risks borne by individuals with intermediate states or declining *Lactobacillus* dominance.

Over the past decade, advancements in detection technologies have driven a significant evolution in vaginal microbiota research. Traditionally, assessments relied primarily on clinical criteria or microscopic scoring methods; while convenient for clinical application, these approaches offer limited resolution regarding microbial community structure and composition [17,18]. With the widespread application of high-throughput sequencing technologies, investigators can now classify the vaginal microbiota into distinct Community State Types (CSTs) based on dominant species and community architecture [7]. Although the potential link between the vaginal microbiota and infertility has garnered considerable attention, existing evidence remains relatively limited and fragmented. While previous meta-analyses suggest that a *Lactobacillus*-dominant state may be associated with a lower risk of infertility [19], the rapid proliferation of sequencing studies necessitates updated, systematic evidence to determine whether distinguishable differences in infertility risk exist across various CSTs, and to quantify the overall impact of a low-*Lactobacillus* vaginal microbiota (LL-VMB) on fertility.

Given these knowledge gaps, this study conducts a systematic review and meta-analysis to comprehensively update the population-based evidence regarding the association between vaginal dysbiosis and female fertility decline. Beyond evaluating the pathogenic risks of traditional dysbiotic states (LL-VMB, BV, and intermediate microbiota), this study further conducts a network meta-analysis based on CSTs. By exploring the specific associations between distinct CSTs and infertility risk, this research aims to provide clearer, evidence-based guidance for identifying infertility risks and developing potential microbiota-targeted interventions.

## 2. Materials and Methods

### 2.1. Study Design and Registration

This systematic review and meta-analysis was conducted in strict accordance with the Preferred Reporting Items for Systematic Reviews and Meta-Analyses (PRISMA 2020) guidelines. The PRISMA checklist is provided as Supplementary Materials S6. The study protocol was registered with PROSPERO (Registration number: CRD420251102003). The research question was formulated using the PICO framework: the population (P) comprised women of reproductive age; the exposure (I) was defined as LL-VMB, BV, intermediate microbiota, or specific CST; the comparator (C) was a *Lactobacillus*-dominant vaginal microbiota; and the outcome (O) was infertility.

### 2.2. Search Strategy

We performed a systematic search of PubMed, Embase, Web of Science, Scopus, and the Cochrane Library from their respective inceptions to 21 July 2025. The search strategy combined Medical Subject Headings (MeSH) and free-text terms, focusing on keywords related to “vaginal microbiota” and “infertility”. The specific search strategy is provided in Supplementary Materials S1.

### 2.3. Inclusion and Exclusion Criteria

Inclusion Criteria: (1) Study Type: observational studies, including cross-sectional, case–control, and cohort studies; (2) Population: women of reproductive age; (3) Exposure Assessment: vaginal microbiota composition assessed using clearly defined and reproducible methods, including Nugent scoring, Amsel/Spiegel clinical criteria, or 16S rRNA gene sequencing; and (4) Outcome: clearly reported infertility outcomes. Data sources included confirmed cases from reproductive medicine centers or clinical records, self-reported data from epidemiological surveys, and infertility observed during follow-up in cohort studies.

Exclusion Criteria: (1) reviews, editorials, conference abstracts, case reports, and animal studies; (2) duplicate publications; (3) studies lacking a fertile or pregnant control group; (4) studies with undefined diagnostic criteria for microbiota composition; (5) non-English publications; and (6) studies assessing the effects of interventions rather than natural exposure states.

### 2.4. Exposure Definitions

LL-VMB: This was defined according to Tamarelle et al. [19,20] as a non-Lactobacillus-dominated vaginal microbial community, identified as meeting any of the following criteria: (1) Nugent score ≥ 4; (2) Amsel/Spiegel-positive; (3) 16S rRNA sequencing indicating a community not dominated by *L. crispatus*, *L. iners*, *L. gasseri*, or *L. jensenii* or explicitly reported as “non-*Lactobacillus* dominant” by the original authors.

BV: BV was defined as a Nugent score of 7–10 or a positive clinical diagnosis using Amsel or Spiegel criteria [18].

Intermediate microbiota: Intermediate microbiota was defined as a Nugent score of 4–6.

CST: CST classification was extracted as reported by the original studies, when available. Consistent with the framework proposed by Ravel et al. [7], classification comprised CST I (*L. crispatus*-dominant), CST II (*L. gasseri*-dominant), CST III (*L. iners*-dominant), CST IV (*Anaerobic*/non-*Lactobacillus*-dominant), and CST V (*L. jensenii*-dominant).

### 2.5. Literature Screening

Title/abstract screening and full-text review were performed independently by two investigators (HY. Z & DY. Q). Disagreements regarding inclusion or exclusion were resolved through consultation with a third investigator.

### 2.6. Data Extraction

Data were extracted and cross-checked independently by two investigators (HY. Z & DY. Q). Extracted variables included: first author, publication year, country, study design, definitions of infertility and control groups, sample sizes, diagnostic methods, number of events, and effect estimates (OR and 95% CI). Adjusted ORs were prioritized; if unavailable, crude ORs were calculated from contingency tables. Corresponding authors were contacted for missing critical data.

To address clinical and classification heterogeneity, infertility outcomes were categorized into three domains: (1) Pregnancy History: primary vs. secondary infertility; (2) Etiology: tubal factor, ovulatory disorders, uterine factors, and unexplained infertility; and (3) Source of Cases: patients recruited from infertility clinics/ART centers vs. women in natural conception cohorts failing to conceive after one year.

Control groups were classified by source: antenatal women, healthy non-pregnant women with a history of pregnancy, healthy non-pregnant women (no history of infertility, pregnancy history unspecified) and healthy non-pregnant women who subsequently became pregnant.

For CST data, when exact numbers were not tabulated but presented in heatmaps, we extracted data based on the cluster groupings and annotations provided in the original figures. Any discrepancies in data extraction were adjudicated by a third investigator (X.H.).

### 2.7. Risk of Bias and Quality Assessment

Quality assessment was performed independently by two investigators (HY. Z & DY. Q). Case–control and cohort studies were evaluated using the Newcastle–Ottawa Scale (NOS) [21], assessing selection (4 stars), comparability (2 stars), and outcome (3 stars). Studies were classified as low risk (Selection 3–4, Comparability 1–2, Outcome 2–3), medium risk (Selection 2, Comparability 1–2, Outcome 2–3), or high risk (Selection 0–1, or Comparability 0, or Outcome 0–1) [22]. Cross-sectional studies were assessed using the Agency for Healthcare Research and Quality (AHRQ) scale [23], with quality categorized as low (0–3), moderate (4–7), or high (8–11). The certainty of evidence for primary outcomes was evaluated using the GRADE approach [24]. Disagreements were resolved by a third investigator (X.H.).

### 2.8. Statistical Analysis

All analyses were performed using R version 4.5.2. The primary effect size was the OR with 95% CI. Effect sizes were pooled on the log scale and back-transformed. For studies with zero events, a continuity correction was applied [25].

For studies reporting CSTs, we utilized the “BUGSnet” package (version 1.1.2) [26] and Just Another Gibbs Sampler (JAGS; version 4.3.2) [27] for Markov Chain Monte Carlo (MCMC) sampling. In the network graph, nodes represented CST types and edges represented direct comparisons, with CST I set as the reference group. MCMC parameters included: 1000 adaptation steps, 1000 burn-in steps, and 10,000 iterations. A random-effects model was used by default to account for anticipated clinical and methodological heterogeneity across studies. CSTs were ranked based on cumulative ranking probabilities. Results are presented as league tables of relative effects, heatmaps, and forest plots.

For the analyses of LL-VMB, BV and intermediate microbiota, heterogeneity was assessed using Cochran’ s Q test (*p* < 0.05 indicating significance) and the *I*^2^ statistic. A random-effects model was used if heterogeneity was present; otherwise, a fixed-effects model was applied. Meta-regression and subgroup analyses were conducted based on country, study design, infertility type, diagnostic method, and study quality. Publication bias was assessed using funnel plots (for comparisons with >10 studies) and Egger’ s test. If asymmetry was detected, the trim-and-fill method was used for adjustment.

## 3. Results

### 3.1. Study Selection

**Figure 1:**
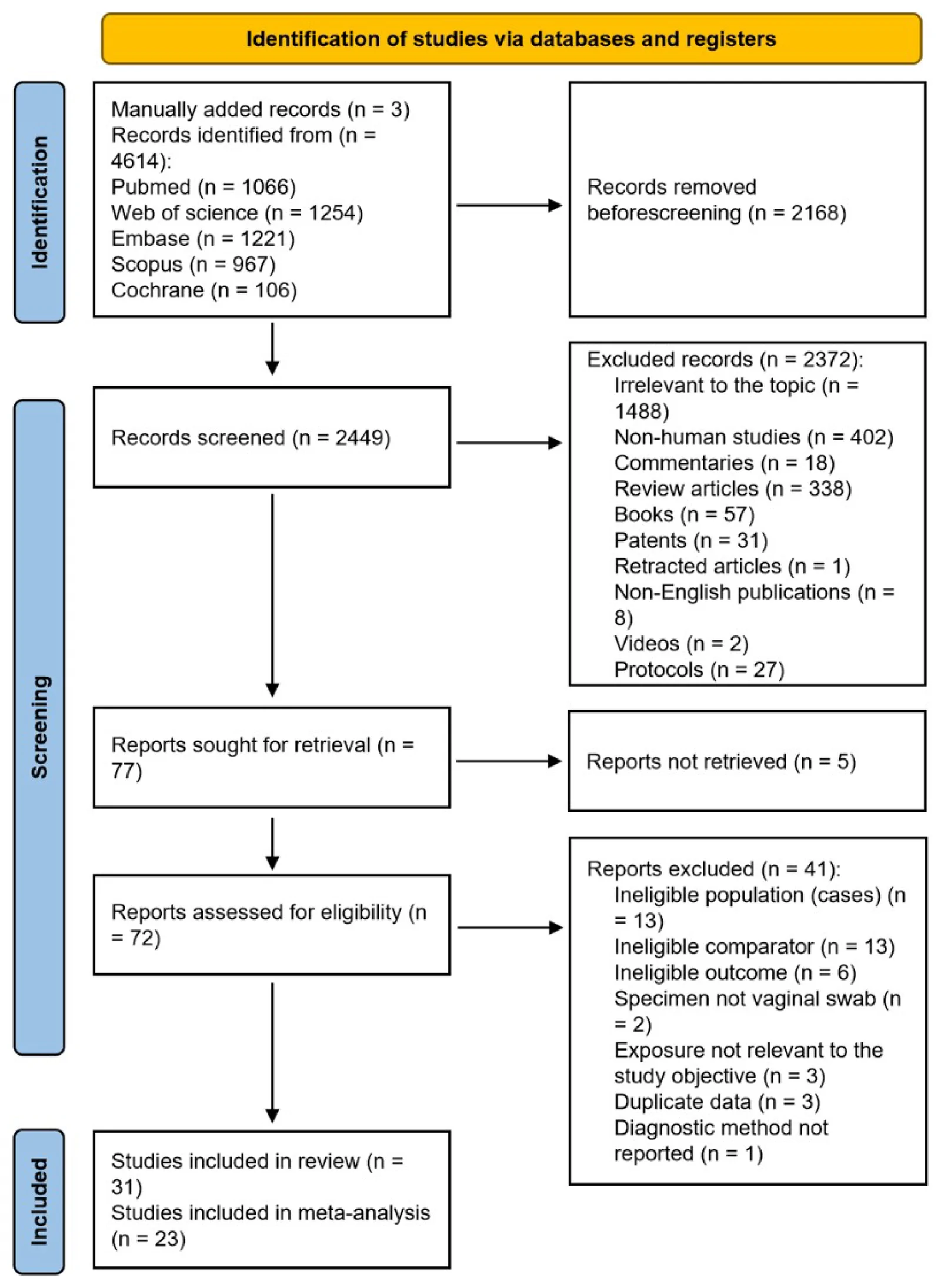
Flow Diagram of Study Selection.

The initial database search yielded 4614 records, and an additional 3 records were identified through manual searching. Following the removal of duplicates, 2449 records remained. After screening titles and abstracts, 72 full-text articles were assessed for eligibility. Ultimately, 31 studies met the inclusion criteria for this systematic review. Of these, 23 provided sufficient data to be included in the quantitative meta-analysis, while the remaining 8 were included only in the qualitative synthesis. The primary reason for exclusion from the meta-analysis was the absence of reported ORs and 95% CIs, which could not be derived from the available raw data. The literature screening process is illustrated in Figure 1.

### 3.2. Study Characteristics and Quality

The 31 included studies were published between 1997 and 2025, comprising a total of 13,060 female participants [16,28,29,30,31,32,33,34,35,36,37,38,39,40,41,42,43,44,45,46,47,48,49,50,51,52,53,54,55,56,57,58] (Table 1 and Table S1). Geographically, the studies covered 16 countries worldwide, with the largest numbers conducted in China and India. Regarding study design, most studies were cross-sectional (n = 24), followed by prospective cohort studies (n = 4) and case–control or nested case–control studies (n = 3). A temporal shift in diagnostic methodology was observed: studies published before 2017 mainly relied on Nugent scoring, Amsel criteria, or Spiegel criteria, whereas studies published from 2017 onward increasingly used molecular methods, including sequencing and PCR-based approaches. Quality assessment using the NOS and AHRQ scales indicated that the overall methodological quality of the included literature was moderate; 6 studies were classified as high quality, 19 as medium quality, and 6 as low quality. Detailed scoring is provided in Tables S2 and S3.

### 3.3. Association Between LL-VMB and Infertility

**Figure 2:**
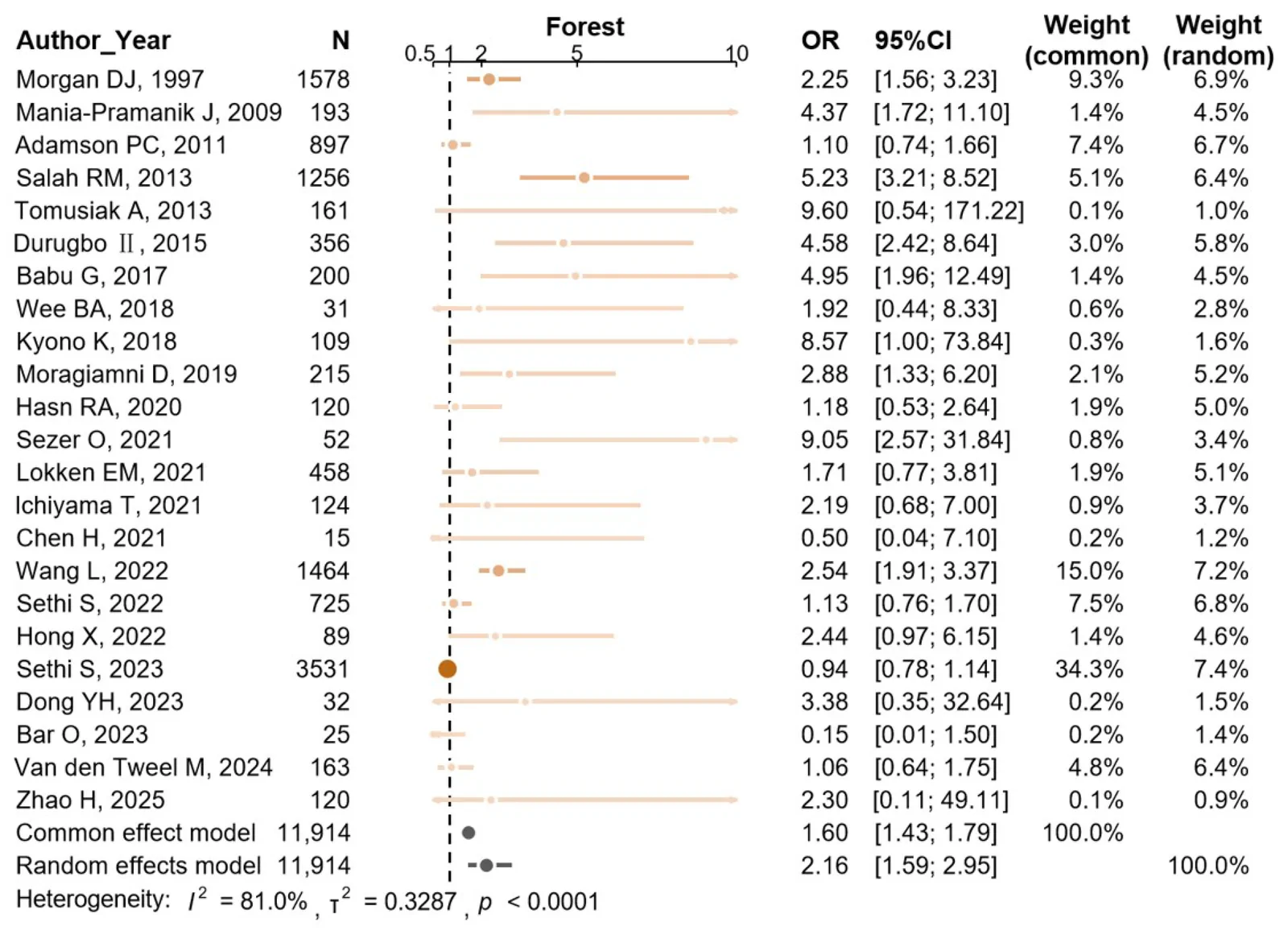
Forest plot of the association between LL-VMB and infertility. The orange markers represent the effect estimates for individual studies, and the horizontal lines represent the corresponding 95% confidence intervals (CIs). The dashed vertical line represents the line of no effect (OR = 1). The gray markers and horizontal lines represent the pooled effect estimates and their 95% CIs. (References: Morgan, D.J., 1997 [16]; Mania-Pramanik, J., 2009 [56]; Adamson, P.C., 2011 [34]; Salah, R.M., 2013 [47]; Tomusiak, A., 2013 [49]; Durugbo, II, 2015 [48]; Babu, G., 2017 [44]; Wee, B.A., 2018 [31]; Kyono, K., 2018 [54]; Moragiamni, D., 2019 [38]; Hasn, R.A., 2020 [58]; Sezer, O., 2021 [59]; Lokken, E.M., 2021 [52]; Ichiyama, T., 2021 [53]; Chen, H., 2021 [55]; Wang, L., 2022 [37]; Sethi, S., 2022 [33]; Hong, X., 2022 [51]; Sethi, S., 2023 [50]; Dong, Y.H., 2023 [28]; Bar, O., 2023 [43]; Van den Tweel, M., 2024 [29]; and Zhao, H., 2025 [39]).

The pooled analysis of 23 studies, comprising a total of 11,914 participants, demonstrated that compared to *Lactobacillus*-dominant vaginal microbiota, LL-VMB was significantly associated with an increased risk of infertility (OR = 2.16, 95% CI: 1.59–2.95) (Figure 2). Heterogeneity testing indicated high inter-study heterogeneity (*I*^2^ = 81.0%, *p* < 0.0001). The certainty of evidence for this outcome was graded as VERY LOW according to GRADE (Table S8).

### 3.4. Association Between BV and Infertility

**Figure 3:**
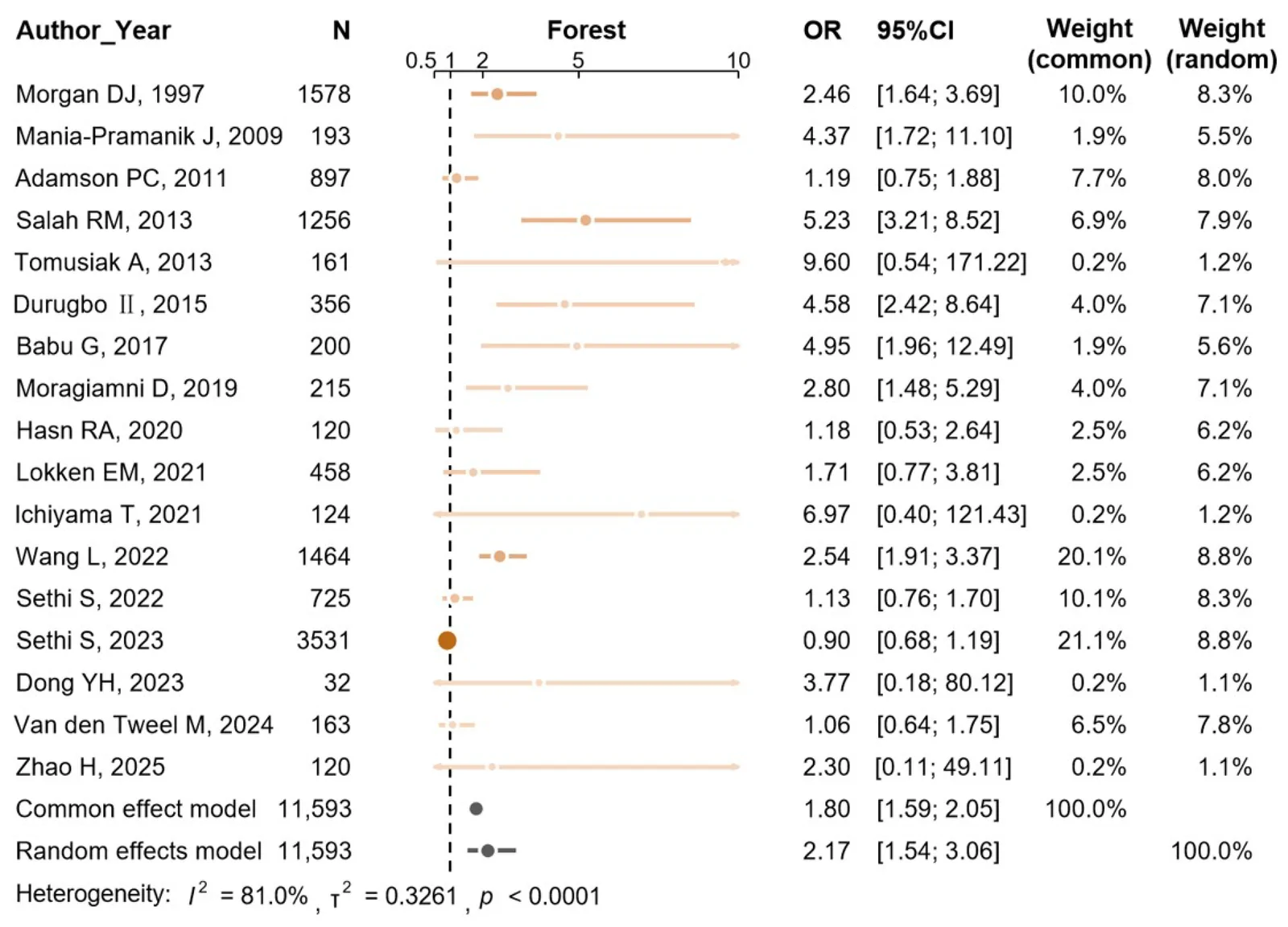
Forest plot of the association between BV and infertility. The orange markers represent the effect estimates for individual studies, and the horizontal lines represent the corresponding 95% confidence intervals (CIs). The dashed vertical line represents the line of no effect (OR = 1). The gray markers and horizontal lines represent the pooled effect estimates and their 95% CIs. (References: Morgan, D.J., 1997 [16]; Mania-Pramanik, J., 2009 [56]; Adamson, P.C., 2011 [34]; Salah, R.M., 2013 [47]; Tomusiak, A., 2013 [49]; Durugbo, II, 2015 [48]; Babu, G., 2017 [44]; Moragiamni, D., 2019 [38]; Hasn, R.A., 2020 [58]; Lokken, E.M., 2021 [52]; Ichiyama, T., 2021 [53]; Wang, L., 2022 [37]; Sethi, S., 2022 [33]; Sethi, S., 2023 [50]; Dong, Y.H., 2023 [28]; Van den Tweel, M., 2024 [29]; and Zhao, H., 2025 [39]).

In the pooled analysis of 17 studies involving 11,593 participants, BV was positively associated with an increased risk of infertility (OR = 2.17, 95% CI: 1.54–3.06) (Figure 3). Similar to LL-VMB, substantial heterogeneity was observed across the included studies *(I*^2^ = 81.0%, *p* < 0.0001). The GRADE certainty of evidence for this outcome was assessed as VERY LOW (Table S9).

### 3.5. Association Between Intermediate Microbiota and Infertility

**Figure 4:**
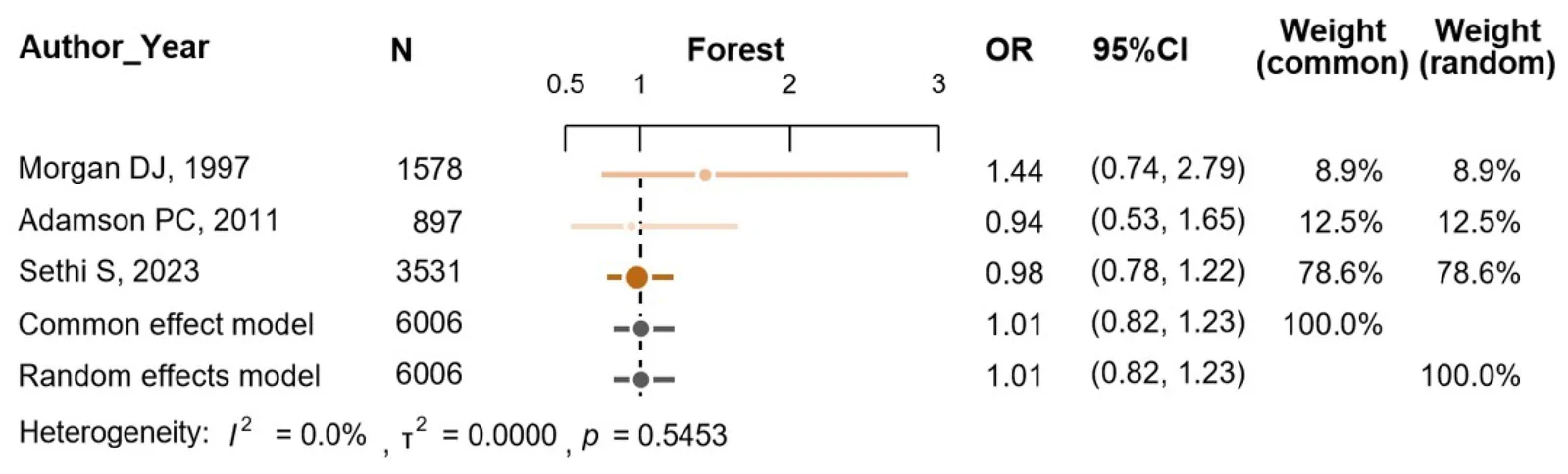
Forest plot of the association between intermediate microbiota and infertility. The orange markers represent the effect estimates for individual studies, and the horizontal lines represent the corresponding 95% confidence intervals (CIs). The dashed vertical line represents the line of no effect (OR = 1). The gray markers and horizontal lines represent the pooled effect estimates and their 95% CIs. (References: Morgan, D.J., 1997 [16]; Adamson, P.C., 2011 [34]; and Sethi, S., 2023 [50]).

A total of 3 studies, encompassing 6006 participants, were included in this pooled analysis. The results indicated no statistically significant association between an intermediate vaginal microbiota state and infertility (*p* > 0.05) (Figure 4). Inter-study heterogeneity was low (*I*^2^ = 0%, *p* > 0.05). The GRADE certainty of evidence for this outcome was evaluated as VERY LOW (Table S10).

### 3.6. Association Between Different CSTs and Infertility

**Figure 5:**
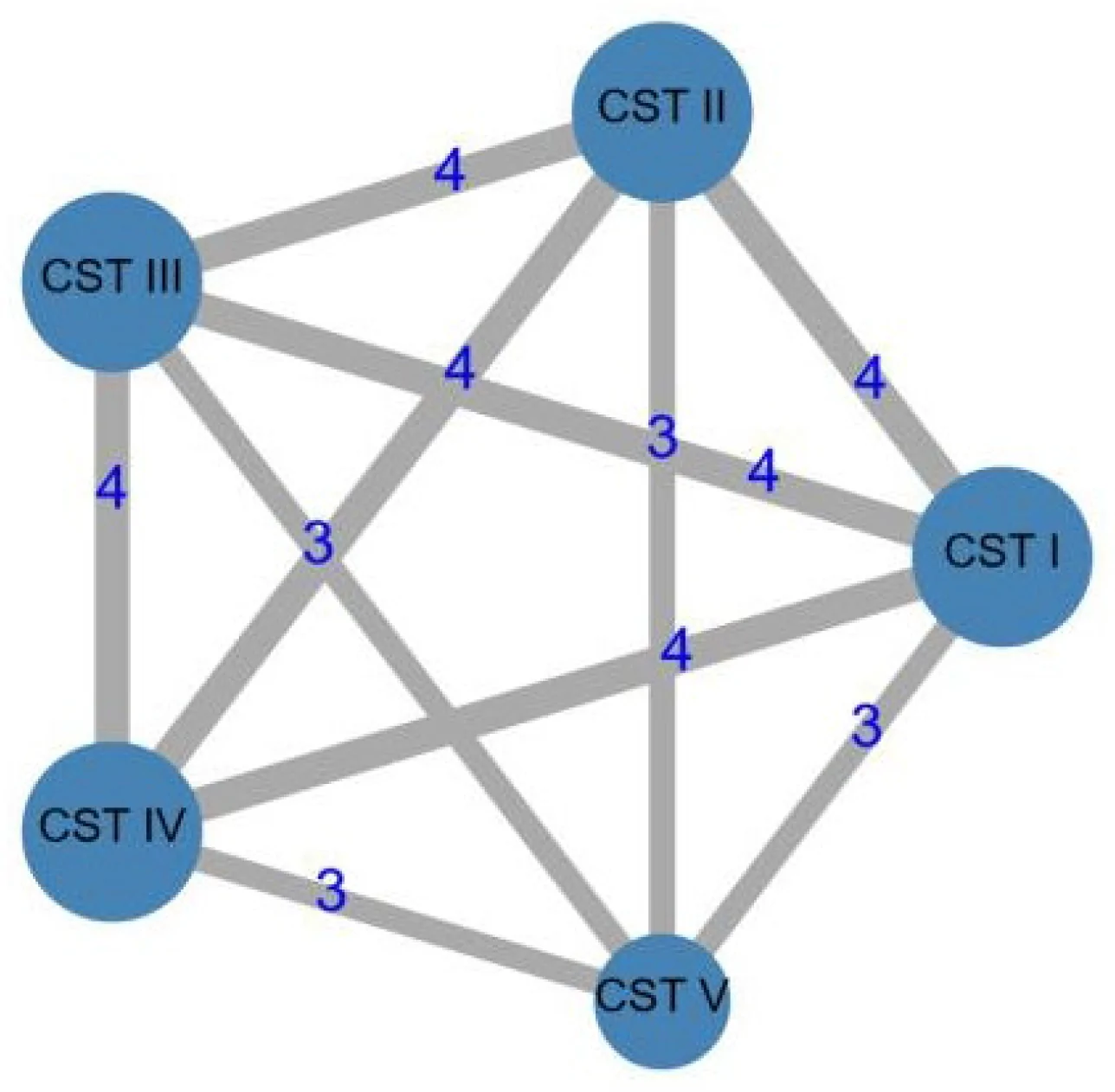
Evidence network plot for CST I–V.

Figure 5 illustrates the direct and indirect comparisons among the different CSTs. The overall network geometry demonstrated robust connectivity, with each pairwise comparison including 3 to 4 studies. Bayesian assessment of global inconsistency showed comparable fit between the consistency and inconsistency models. The DIC values were 34.10 and 33.53, respectively, and the corresponding posterior mean residual deviances were 21.73 and 21.11. The small DIC difference (ΔDIC = −0.57) indicated that the inconsistency model did not provide a meaningful improvement in fit. Therefore, no clear evidence of global inconsistency was identified, although the sparse evidence network warrants cautious interpretation. Furthermore, there was no clear evidence of disagreement between the direct and indirect estimates for the evaluable comparisons.

**Figure 6:**
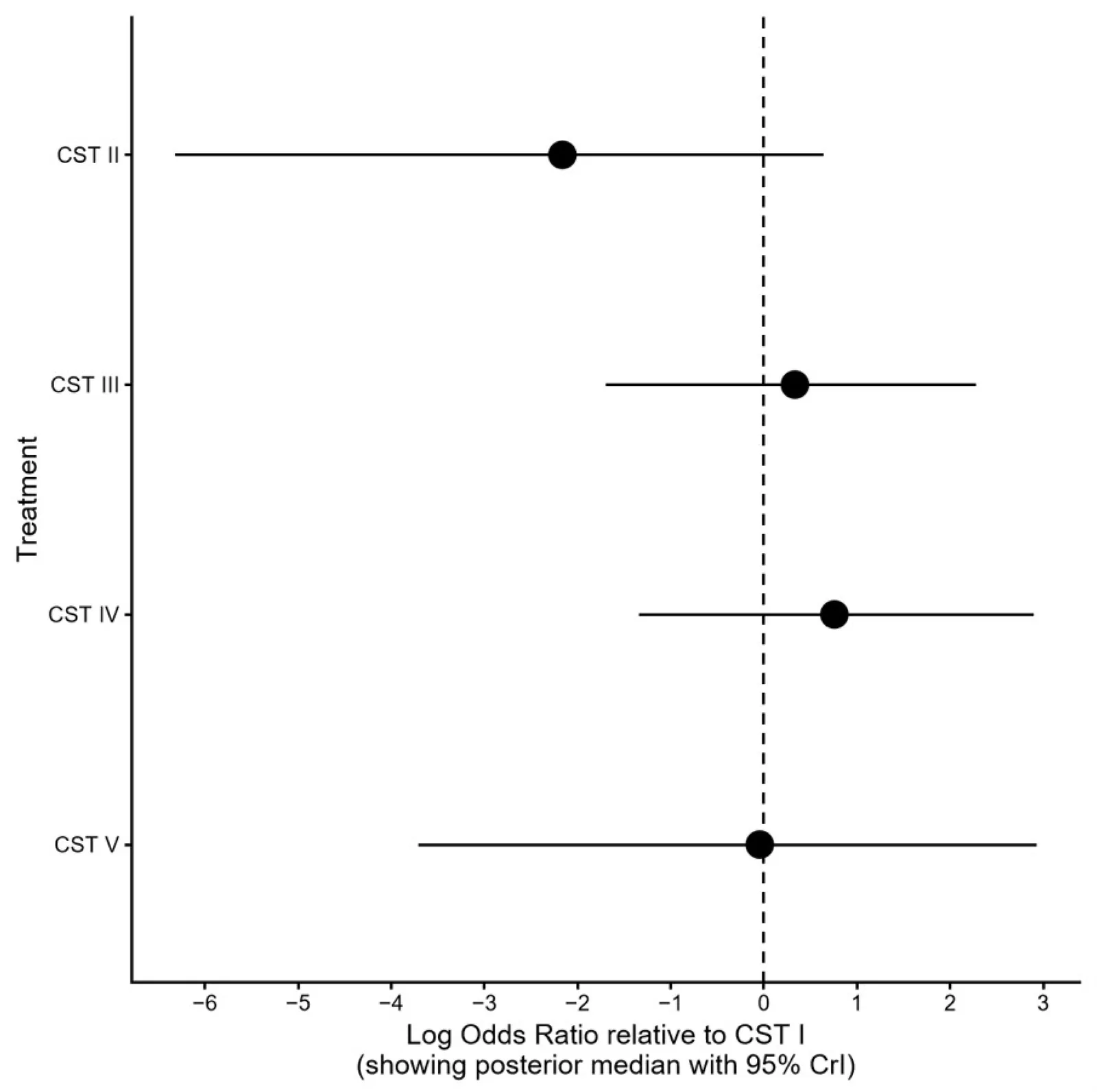
Forest Plot of the Association Between CSTs and Infertility (Reference: CST I). References: Wee, B.A., 2018 [31]; Dong, Y.H., 2023 [28]; Bar, O., 2023 [43]; and Van den Tweel, M., 2024 [29].

**Figure 7:**
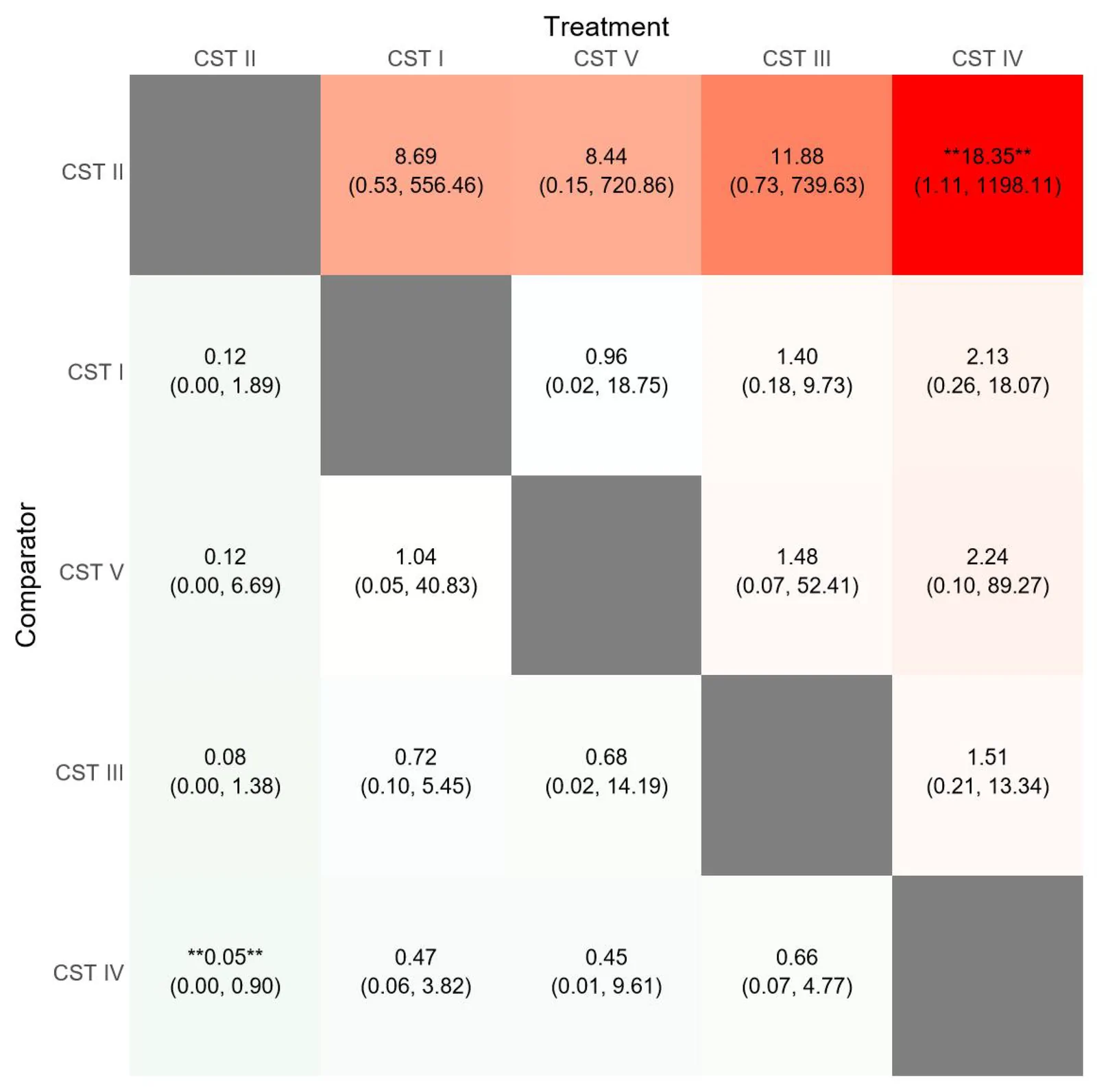
League table of the associations between CST subtypes and infertility risk. Red-shaded cells indicate ORs > 1, whereas green-shaded cells indicate ORs < 1; darker shading indicates a greater deviation from the null value (OR = 1). ** indicates comparisons for which the 95% CrI does not include 1.

**Figure 8:**
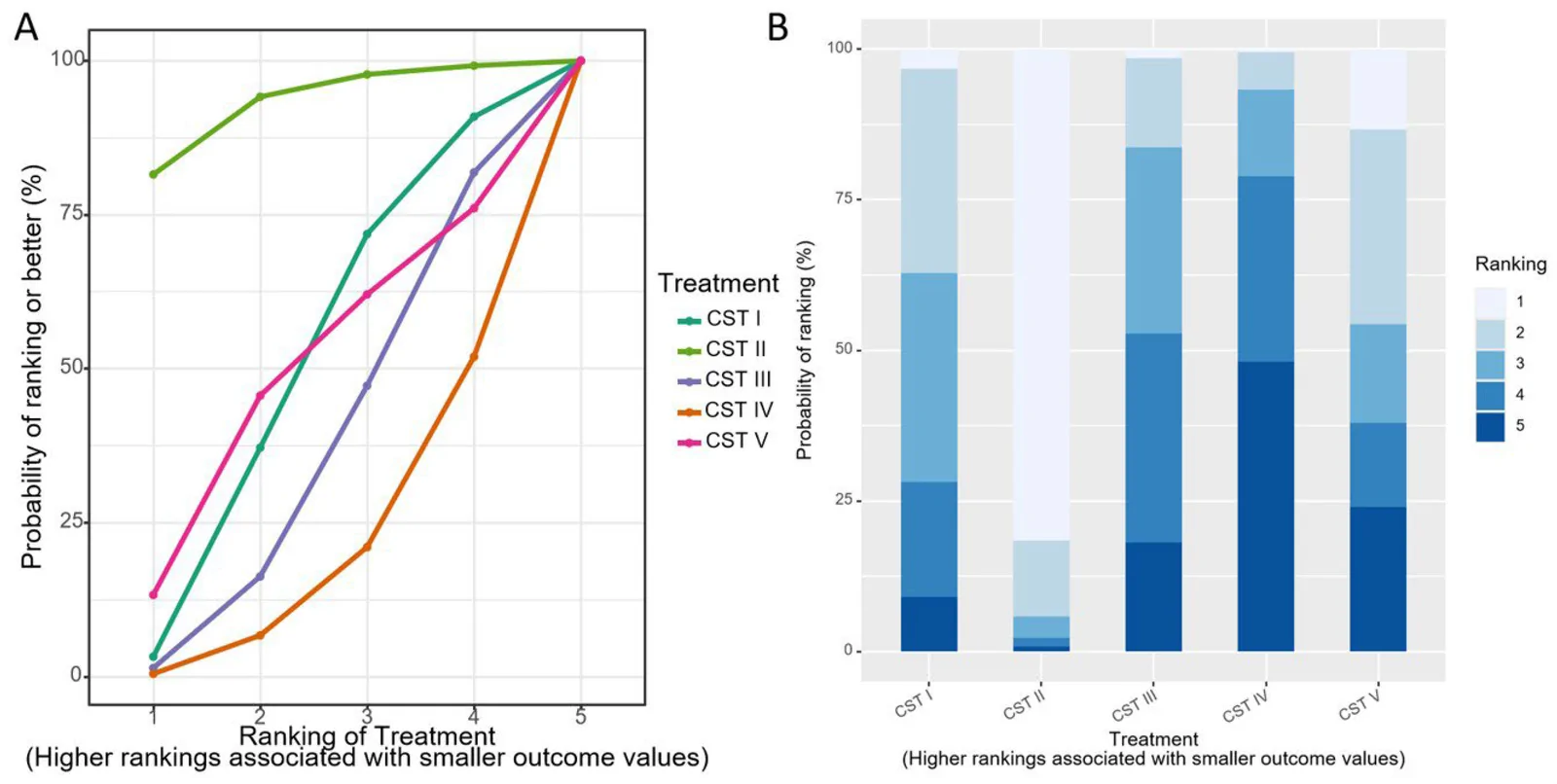
Cumulative Ranking Probability Plot for the Association Between CSTs and Infertility (**A**,**B**).

Using CST I as the reference group, none of the other individual CSTs demonstrated a statistically significant association with infertility (*p* > 0.05) (Figure 6). The league table additionally presents all possible pairwise comparisons estimated within the network. Among these secondary comparisons, CST IV was associated with a significantly higher risk of infertility when compared to CST II (OR = 18.35, 95% CrI: 1.11–1198.11) (Figure 7). However, this result is highly imprecise due to severe data sparsity. Comparisons among the remaining CST types yielded no statistically significant differences (*p* > 0.05). Cumulative ranking probabilities suggested that CST II was the most likely to be ranked as the lowest risk state, whereas CST IV was the most likely to be ranked as the highest risk state (Figure 8). These findings suggest that the risk of infertility may vary distinctly across different CSTs. The GRADE evaluations for all CST comparisons were VERY LOW (Table S11).

### 3.7. Sensitivity Analysis

Given the substantial heterogeneity observed in the associations between LL-VMB, BV, and infertility, meta-regression analyses were conducted incorporating country, study design, infertility type, control group type, diagnostic method, and study quality as covariates. The results indicated that none of the regression coefficients for these variables reached statistical significance (*p* > 0.05) (Tables S4 and S5).

Subgroup analyses were conducted according to country, study type, type of infertility, type of control, diagnostic method, and study quality. For LL-VMB, significant positive associations with infertility were observed in most subgroups. However, the association was not statistically significant in studies conducted in India, in nested case–control and case–control studies, in the primary infertility and unexplained infertility subgroups, or in studies using sequencing-based diagnostic methods.

For BV, subgroup analyses showed broadly consistent positive associations with infertility. However, the association was not statistically significant in studies conducted in India, in nested case–control and case–control studies, or in the subgroup including other infertility types (Tables S6 and S7).

Sensitivity analyses were performed using the leave-one-out method to assess the robustness of the pooled estimates. The results remained stable after sequentially omitting individual studies. These findings suggest that the overall results were not driven by any single study.

### 3.8. Publication Bias

**Figure 9:**
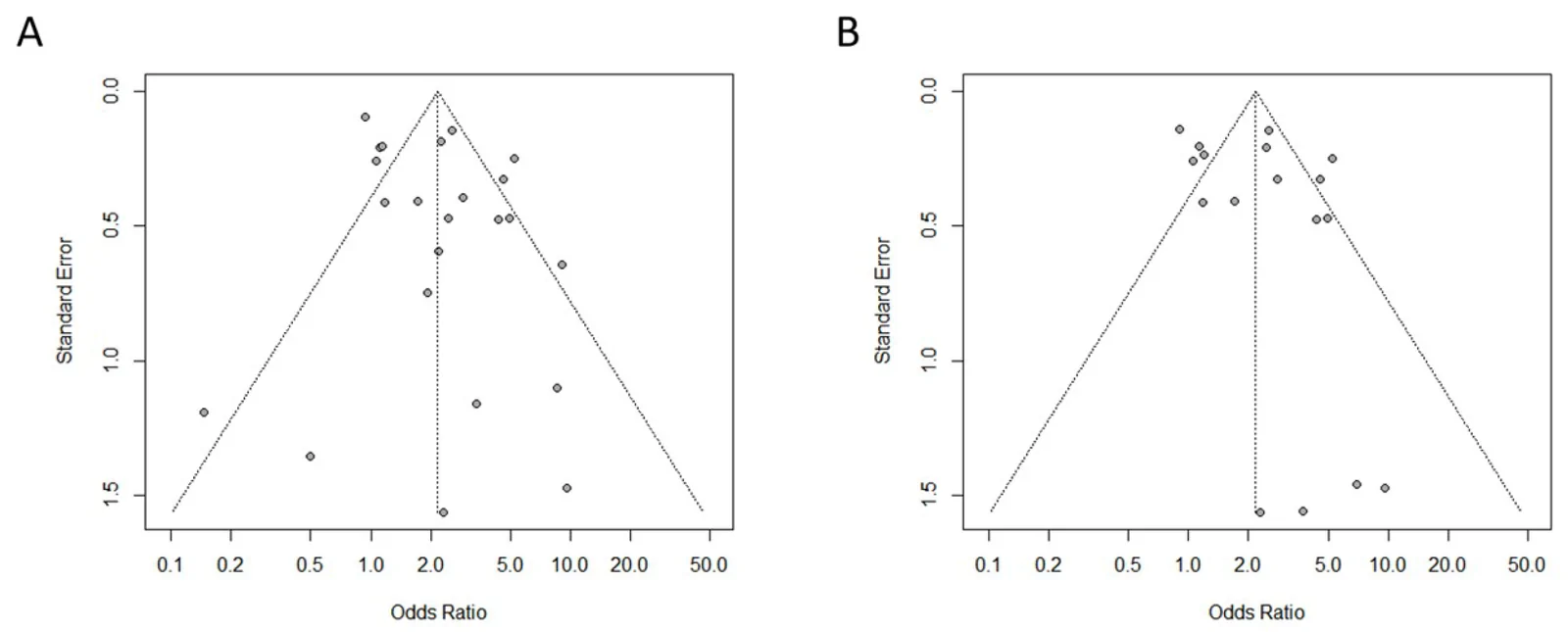
Funnel plots for publication bias. (**A**) Funnel plot for the risk of infertility associated with LL-VMB. (**B**) Funnel plot for the risk of infertility associated with BV.

**Figure 10:**
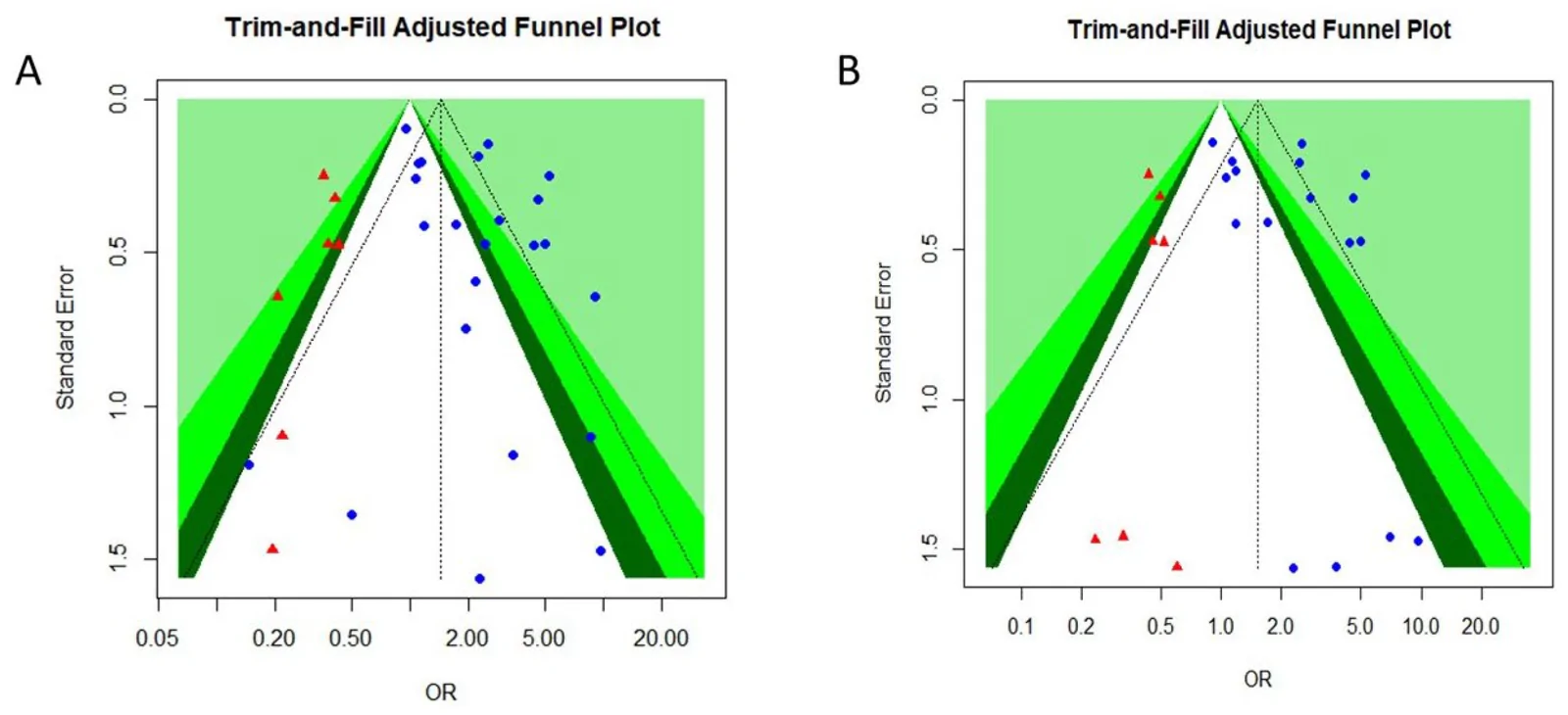
Funnel Plots Adjusted by the Trim-and-Fill Method. (**A**) Adjusted funnel plot for the association between LL-VMB and infertility risk. (**B**) Adjusted funnel plot for the association between BV and infertility risk. Blue circles represent the observed studies, whereas red triangles represent studies imputed using the trim-and-fill method. The white region represents *p* > 0.10; the dark-green region represents 0.05 < *p* ≤ 0.10; the green region represents 0.01 < *p* ≤ 0.05; and the light-green region represents *p* ≤ 0.01.

Visual inspection of the funnel plots for LL-VMB and BV did not reveal overt asymmetry (Figure 9). Egger’s test yielded a borderline non-significant result (t = 1.99, *p* = 0.0594), and Begg’s rank correlation test also indicated no significant asymmetry (z = −0.29, *p* = 0.7714). To further assess the potential impact of small study effects, a trim-and-fill analysis was performed. The adjusted pooled effect size after trim-and-fill correction was OR = 1.44 (95% CI: 0.98–2.11). Egger’ s test for BV did not detect a risk of bias (t = 1.43, *p* = 0.1732), Begg’s rank correlation test also indicated no significant asymmetry (z = 0.33, *p* = 0.7417), and the adjusted pooled effect size after trim-and-fill correction was OR = 1.52 (95% CI: 1.04–2.22). The trim-and-fill-adjusted estimate for BV remained statistically significant. In contrast, the adjusted LL-VMB estimate was attenuated, suggesting that the LL-VMB result may be sensitive to potential small-study effects (Figure 10).

## 4. Discussion

Substantial evidence indicates that the unique female vaginal ecosystem plays a pivotal role in reproductive health. This study analyzed the association between the vaginal microbiota and infertility risk in women of reproductive age. Our findings are generally consistent with previous research, suggesting that an abnormal vaginal microbiota is associated with an elevated risk of infertility [19]. Specifically, LL-VMB was associated with an increased risk of infertility, and BV was similarly positively correlated with infertility. In contrast, no statistically significant association was observed between intermediate microbiota and infertility; however, this conclusion relies on only three studies representing limited evidence, necessitating further verification through larger, prospective cohorts. With the rapid advancement of sequencing technologies, vaginal microbiota research has progressively transitioned from clinical criteria to classification systems based on community structure. This study further explored analyses based on distinct CSTs and, to our knowledge, is the first to integrate the relationships between different CSTs and infertility using a network meta-analysis. The results suggest that CST IV is associated with a significantly higher risk of infertility compared to CST II, thereby more comprehensively expanding our understanding of the association between vaginal dysbiosis and female infertility.

Although the exact molecular mechanisms by which vaginal dysbiosis leads to infertility have not been fully elucidated, current evidence suggests that the increased risk associated with LL-VMB and BV observed in this study can be attributed to four primary pathways: First, the chronic inflammation hypothesis. LL-VMB and BV are frequently accompanied by elevated vaginal pH, compromised mucosal barrier function, and enhanced local inflammatory responses [10,60,61]. While local vaginal inflammation may not directly damage oocytes, recurrent and persistent dysbiosis may promote the ascension of inflammatory signals to the cervix, endometrium, and the tubal microenvironment. This can induce occult pelvic inflammatory processes, leading to scarring and adhesions, and ultimately resulting in tubal obstruction—a major etiology of female infertility [62,63]. Second, sperm toxicity and fertilization impairment. Existing studies indicate that BV-associated anaerobes produce various inflammatory mediators and toxic metabolites that may interfere with sperm capacitation, induce premature acrosome reaction, and significantly inhibit sperm motility, thereby reducing fertilization efficiency during natural conception [64,65,66]. Third, the endocrine disruption hypothesis. Vaginal dysbiosis may disrupt the hypothalamic–pituitary–ovarian axis, contributing to hormonal imbalance and impaired follicular development or endometrial receptivity [67,68]. Fourth, increased susceptibility to reproductive tract infections. A *Lactobacillus*-dominant vaginal microbiota maintains an acidic environment (pH 3.5–4.5) by metabolizing glycogen into lactic acid, thereby inhibiting pathogen growth and maintaining barrier homeostasis [69,70]. When *Lactobacillus* abundance declines and vaginal pH rises, conditions become favorable for the proliferation of opportunistic pathogens [69]. This increased susceptibility not only renders the host more vulnerable to invasion by reproductive tract pathogens, but the resulting immune cascade may further impair endometrial receptivity, leading to adverse reproductive outcomes [71,72,73,74].

With the development of high-throughput sequencing technologies, researchers are now able to conduct more granular taxonomic analyses of the vaginal microbiota. The conventional clinical classification defines LL-VMB as communities not dominated by any of the four species: *L. crispatus, L. gasseri*, *L. jensenii*, or *L. iners*. However, this grouping biologically equates the highly protective *L. crispatus*-dominant state with the transitional and functionally distinct *L. iners*-dominant state, potentially underestimating the risk of dysbiosis-related outcomes. The CST classification system proposed by Ravel et al. categorizes the vaginal microbiota into five distinct groups based on dominant bacterial species and community structure: CST I (*L. crispatus*-dominant), CST II (*L. gasseri*-dominant), CST III (*L. iners*-dominant), CST V (*L. jensenii*-dominant), and CST IV, which is characterized by a high diversity of predominantly anaerobic bacteria and a depletion of *Lactobacilli* [7,75]. CST I is generally considered highly protective, producing both D-lactic acid and hydrogen peroxide, which strongly suppress pathogen growth [76]. CST II and CST V also produce hydrogen peroxide, though their ecological roles are less characterized compared to CST I [76]. CST III is functionally unique: *L. iners* has a smaller genome and lacks hydrogen peroxide production [76,77]. It is often regarded as a transitional species that can rapidly shift to a dysbiotic community under inflammatory or hormonal changes [77]. In contrast, CST IV represents a high diversity, non-Lactobacillus-dominant state characterized by elevated pH, increased inflammation, and adverse reproductive outcomes. These functional differences among CSTs directly influence microbial associations, defined as co-occurrence or co-exclusion patterns between taxa. Building upon this methodological evolution, the present study is the first to employ a network meta-analysis to integrate direct and indirect evidence regarding the associations between different CST subtypes and infertility. Our analysis revealed that compared to the *L. gasseri*-dominant CST II, CST IV was associated with a significantly elevated risk of infertility. Although the wide confidence interval warrants cautious interpretation, the directionality of the effect size carries significant clinical implications. This finding is highly consistent with recent research trends: as a classic molecular signature of vaginal dysbiosis, CST IV has been repeatedly linked to tubal factor infertility, in vitro fertilization (IVF) implantation failure, and early miscarriage [19,69,78]. This also corroborates our overarching conclusions regarding LL-VMB and BV, suggesting that a microbial community structure characterized by *Lactobacillus* depletion and anaerobic dominance is a core microecological feature detrimental to female fertility. In contrast, risk assessments for *Lactobacillus*-dominant states such as CST I, III, and V remain heterogeneous across different studies, underscoring the need for future large-scale sequencing studies to clarify their stratified risks. In summary, the identification of a CST IV microbial profile during clinical screening warrants heightened attention. This subtype indicates a reproductive tract microenvironment that is hostile to conception and should be prioritized as a key target for future infertility interventions and microecological modulation.

Despite the significant findings of this study, several limitations warrant consideration. First, the majority of the included primary studies were cross-sectional in design, which inherently precludes the establishment of causal relationships between vaginal dysbiosis and infertility. Second, considerable heterogeneity was observed across the included literature. This meta-analysis encompassed diverse study populations and utilized varying diagnostic criteria; some studies defined BV using Nugent scoring or culture methods, whereas others employed 16S rRNA sequencing to classify microbiota profiles. Furthermore, the definition of infertility itself was not uniformly reported across studies: some focused exclusively on failure to conceive, while others may have included early pregnancy loss or other conditions. Similarly, the definition of control groups also varied with respect to pregnancy history, parity, and the degree of fertility confirmation, and this information was often incompletely reported in the original studies. These inconsistencies in participant classification further contributed to heterogeneity. These discrepancies in taxonomic definitions and experimental methodologies inevitably increase the uncertainty of the pooled analyses. Although meta-regression was conducted to explore the sources of heterogeneity, it remains challenging to completely eliminate the potential confounding effects of unmeasured variables. Third, non-standardized data reporting formats in some primary studies prevented their inclusion in our quantitative synthesis, leading to potential information loss.

In light of these limitations, further high-quality research is imperative to deepen our understanding of the relationship between the vaginal microbiota and female infertility. Current evidence relies heavily on cross-sectional data, yielding a very low certainty of evidence in GRADE. Future research must prioritize high-quality prospective interventional studies. By implementing targeted microbiota interventions in reproductive-aged women with vaginal dysbiosis and evaluating whether microbial restoration reduces the incidence of unexplained infertility, researchers can provide high-level, evidence-based validation for the vaginal microbiota as a viable therapeutic target. Furthermore, standalone microbiome profiling primarily describes community structure without fully capturing the functional state of the microenvironment. Future investigations should integrate multi-omics technologies, including metabolomics and proteomics, to construct robust predictive models. This multi-omics approach will enhance the accuracy of infertility screening and provide a molecular foundation for clinical diagnosis and efficacy evaluation. Finally, we strongly advocate for the establishment of standardized reporting guidelines in vaginal microecology research. Comprehensive disclosure of microbiota data, including the relative abundance profiles of major taxa for all study subjects, will facilitate accurate cross-study comparisons and meta-analytical integration, ultimately enabling the definitive validation of the long-term impacts of microbial profiles on fertility utilizing larger-scale datasets.

## 5. Conclusions

In conclusion, the findings of this study demonstrate a strong association between vaginal microbiota composition and female fertility. LL-VMB and BV were associated with higher odds of infertility, whereas the evidence for differences among individual CST subtypes remained inconclusive. Exploratory ranking results suggested that CST IV may represent a relatively higher-risk profile, but this finding requires confirmation in larger sequencing-based studies. Although a definitive causal link has yet to be fully established given the observational nature and heterogeneity of existing literature, our results underscore the critical role of maintaining vaginal microbiota homeostasis in reproductive health. These findings suggest that the vaginal microbiota holds promise as a novel biomarker for fertility assessment, providing a robust scientific basis for elucidating the etiology of infertility and guiding future strategies for microecological intervention.

## Figures and Tables

**Table 1:** Characteristics of Included Studies.

Year	First Author	Study Design	Country	Type of Infertility	Type of Control Group	Age (Years, Mean ± SD or Range)	Diagnostic Method	N	Quality
1997	Morgan, D.J. [16]	Cross-section	UK	6	a	Not reported	Nugent	1578	Low
2009	Mania-Pramanik, J. [56]	Cross-section	India	6	c	19–47	Nugent	193	Medium
2011	Adamson, P.C. [34]	Nest case–control	India	1	c	Inf: 24.00 ± 3.40 Con: 26.10 ± 3.00	Nugent	897	Low
2013	Salah, R.M. [47]	Cross-section	Egypt	6 (3 + 4 + 5)	c	Inf: 27.10 ± 2.20 Con: 25.80 ± 3.20	Spiegel	1256	High
2013	Tomusiak, A. [49]	Cross-section	Poland	6	b	20–40	Nugent	161	Medium
2015	Durugbo, II [48]	Cross-section	Nigeria	3	c	22–36	Amsel	356	Medium
2017	Babu, G. [44]	Cross-section	India	6 (1 + 2)	c	18–45	Nugent	200	Medium
2017	Campisciano, G. [57]	Cross-section	Italy	6	c	32–40	PCR	96	Medium
2018	Wee, B.A. [31]	Case–control	Australia	6	c	Inf: 37.6 (28–45) Con: 42.75 (35–48)	Sequencing + PCR	31	Medium
2018	Kyono, K. [54]	Cross-section	Japan	6	c	Inf: 36.99 ± 4.22, 33.22 ± 3.64 Con: 36.57 ± 6.63	Sequencing + PCR	109	Medium
2019	Moragiamni, D. [38]	Cross-section	Greece	6	b	Inf: 31.98 (22–45) Con: 44.00 (22–45)	Nugent + Amsel	215	Medium
2020	Hasn, R.A. [58]	Cross-section	Iraq	6 (5)	c	18–45	Amsel	120	Medium
2020	Zhao, C. [45]	Cross-section	China	2	c	Inf: 30.31 ± 6.76, 31.12 ± 5.02 Con: 31.11 ± 7.05, 30.97 ± 7.61	Sequencing	122	Medium
2021	Sun, N. [42]	Cross-section	China	6	c	Not reported	Sequencing + PCR	184	Medium
2021	Azpiroz, M.A. [35]	Cross-section	USA	5	c	27–52	Sequencing + PCR	307	Medium
2021	Sezer, O. [59]	Cross-section	Turkey	6 (5)	b	Inf: 30.20 ± 7.10 Con: 30.40 ± 6.60	PCR	52	High
2021	Lokken, E.M. [52]	Prospective Cohort	Kenyan	7	d	29 (25–34)	Nugent	458	High
2021	Ichiyama, T. [53]	Cross-section	Japan	6	c	Inf: 38.3 ± 4.2 Con: 32.0 ± 4.0	Nugent + Sequencing	124	High
2021	Chen, H. [55]	Cross-section	USA	6 (3)	c	Inf: 33.9 ± 4.2 Con: 33.6 ± 3.7	Sequencing	15	High
2021	Manzoor, A. [46]	Cross-section	Pakistani	6	c	28.25 ± 5.47	Sequencing + PCR	32	Medium
2022	Patel, N. [40]	Cross-section	India	6	c	Inf: 34.50 ± 4.80, 30.80 ± 3.42 Con: 27.90 ± 3.80	Sequencing	31	Medium
2022	Wang, L. [37]	Cross-section	China	6	c	Inf: 28.55 ± 3.16 Con: 30.13 ± 4.47	Spiegel	1464	Medium
2022	Sethi, S. [33]	Cross-section	India	6 (1 + 2)	c	>18 (Mean 31.71)	Nugent	725	Medium
2022	Hong, X. [51]	Prospective Cohort	China	7	d	Inf: 29.52 ± 3.73 Con: 27.98 ± 2.67	PCR	89	Low
2023	Sethi, S. [50]	Cross-section	India	6	c	28.00 (15.00–50.00)	Nugent	3531	Medium
2023	Dong, Y.H. [28]	Cross-section	China	5	b	Inf: 31.40 ± 4.70 Con: 33.80 ± 4.50	PCR	32	Medium
2023	Bar, O. [43]	Cross-section	USA	6	c	Not reported	Sequencing	25	Low
2024	Van den Tweel M., [29]	Prospective Cohort	Holland	7	d	33.73 (18.00–43.00)	PCR	163	High
2024	Chopra, C. [30]	Prospective Cohort	India	6	d	Inf: 26.12 ± 4.03 Con: 25.32 ± 6.50	Sequencing	80	Low
2025	Chen, X. [36]	Cross-section	China	6	b	Inf: 31.7 ± 4.8 Con: 32.3 ± 4.52	Sequencing	294	Medium
2025	Zhao, H. [39]	Case–control	China	6 (3 + 4)	c	Inf: 31.76 ± 4.68, 29.18 ± 4.36 Con: 32.92 ± 5.22	Sequencing	120	Low

Note: Inf: Infertility Group; Con: Control Group; SD: Standard Deviation; PCR: Polymerase Chain Reaction. Infertility Type: (1) primary infertility; (2) secondary infertility; (3) tubal factor infertility; (4) ovulatory disorders; (5) unexplained infertility; (6) clinical population from infertility clinics or undergoing ART/IVF/ICSI; (7) natural conception cohorts: participants who failed to conceive after follow-up. Control Group Type: (a) antenatal women; (b) healthy non-pregnant women with a history of pregnancy; (c) healthy non-pregnant women (no history of infertility, pregnancy history unspecified); (d) healthy non-pregnant women who subsequently became pregnant.

## Data Availability

The original contributions presented in this study are included in the article and Supplementary Materials. Further inquiries can be directed to the corresponding author.

## Supplementary Materials

The following supporting information can be downloaded at: https://www.mdpi.com/article/10.3390/microorganisms14092094/s1, Supplementary Material S1: Search Strategies; Supplementary Material S2: Characteristics of Included Studies, including Table S1: Characteristics of Included Studies; Supplementary Material S3: Quality Assessment, including Table S2: Quality Assessment of Cross-Sectional Studies (AHRQ Scale) and Table S3: Quality Assessment of Analytical Studies (NOS Scale); Supplementary Material S4: Sensitivity Analysis, including Table S4: Meta-Regression Analysis of the Association Between LL-VMB and Infertility, Table S5: Meta-Regression Analysis of the Association Between BV and Infertility, Table S6: Subgroup Analysis of the Association Between LL-VMB and Infertility, and Table S7: Subgroup Analysis of the Association Between BV and Infertility; Supplementary Material S5: GRADE Assessments, including Table S8: GRADE Assessment for the Association Between LL-VMB and Infertility, Table S9: GRADE Assessment for the Association Between BV and Infertility, Table S10: GRADE Assessment for the Association Between Intermediate Microbiota and Infertility, and Table S11: GRADE Assessment for the Association Between CSTs and Infertility; Supplementary Material S6: PRISMA 2020 Checklist. Ref. [79] can be found in Supplementary Materials.

## Abbreviations

WHO	World Health Organization
BV	Bacterial vaginosis
PID	Pelvic inflammatory disease
ART	Assisted reproductive technology
CST	Community State Types
CST I	*L. crispatus*-dominant
CST II	*L. gasseri*-dominant
CST III	*L. iners*-dominant
CST IV	*Anaerobic*/non-*Lactobacillus*-dominant
CST V	*L. jensenii*-dominant
LL-VMB	Low-*Lactobacillus* vaginal microbiota
MeSH	Medical Subject Headings
NOS	Newcastle–Ottawa Scale
AHRQ	Agency for Healthcare Research and Quality
MCMC	Markov Chain Monte Carlo
JAGS	Just Another Gibbs Sampler
IVF	In vitro fertilization
ICSI	Intracytoplasmic sperm injection
