# Supplementary material for: Association Between Vaginal Microbiota and Female Infertility: A Systematic Review and Meta-Analysis
Source: Microorganisms. 2026 Sep 18;14(9):2094. doi: 10.3390/microorganisms14092094 (PMC13608893; doi:10.3390/microorganisms14092094)
Supplement: Supplementary file 1 [file microorganisms-14-02094-s001.zip › microorganisms-4453058-supplementary.pdf]

Supplementary Material S1. Search Strategies

Pubmed

("Infertility"[MeSH Terms] OR ("infertiles"[All Fields] OR "infertilities"[All Fields] OR "Infertility"[MeSH Terms] OR "Infertility"[All Fields] OR "infertile"[All Fields] OR "infertility s"[All Fields]) OR ("Infertility"[MeSH Terms] OR "Infertility"[All Fields] OR "sterile"[All Fields] OR "sterility"[All Fields] OR "sterilant"[All Fields] OR "sterilants"[All Fields] OR "sterilely"[All Fields] OR "steriles"[All Fields] OR "sterilisation"[All Fields] OR "sterilization, reproductive"[MeSH Terms] OR ("sterilization"[All Fields] AND "reproductive"[All Fields]) OR "reproductive sterilization"[All Fields] OR "sterilization"[All Fields] OR "sterilization"[MeSH Terms] OR "sterilisations"[All Fields] OR "sterilised"[All Fields] OR "steriliser"[All Fields] OR "sterilisers"[All Fields] OR "sterilising"[All Fields] OR "sterilities"[All Fields] OR "sterilise"[All Fields] OR "sterilize"[All Fields] OR "sterilizations"[All Fields] OR "sterilized"[All Fields] OR "sterilizer"[All Fields] OR "sterilizers"[All Fields] OR "sterilizes"[All Fields] OR "sterilizing"[All Fields]) OR ("fertiles"[All Fields] OR "fertility"[MeSH Terms] OR "fertility"[All Fields] OR "fertile"[All Fields] OR "fertilities"[All Fields]) OR ("Infertility"[MeSH Terms] OR "Infertility"[All Fields] OR "subfertility"[All Fields] OR "subfertile"[All Fields])) AND ("vaginitis"[MeSH Terms] OR ("vagina"[MeSH Terms] OR "vagina"[All Fields] OR "vaginal"[All Fields] OR "vaginally"[All Fields] OR "vaginals"[All Fields] OR "vaginitis"[MeSH Terms] OR "vaginitis"[All Fields] OR "vaginitides"[All Fields]) OR ("genitalia"[MeSH Terms] OR "genitalia"[All Fields] OR "genital"[All Fields] OR "genitals"[All Fields] OR "genitally"[All Fields]) OR "vagin*"[All Fields]) AND ("Microbiota"[MeSH Terms] OR "microbiot*"[All Fields] OR "microfilm*"[All Fields] OR "microbiom*"[All Fields] OR ("flora"[All Fields] OR "florae"[All Fields] OR "floras"[All Fields]) OR "microflor*"[All Fields] OR ("dysbiosis"[MeSH Terms] OR "dysbiosis"[All Fields] OR "dysbioses"[All Fields]))

Embase

('infertility'/exp OR infertility OR sterility OR fertility OR subfertility)

AND ('vaginitis'/exp OR vaginitis OR genital OR vagin*)

AND ('microbiota'/exp OR microbiot* OR microfilm* OR microbiom* OR flora OR microflor* OR dysbiosis)

Web of science

TS=(infertility OR sterility OR fertility OR subfertility)

AND TS=(vaginitis OR genital OR vagin*)

AND TS=(microbiot* OR microfilm* OR microbiom* OR flora OR microflor* OR

dysbiosis)

Scopus

TITLE-ABS-KEY(infertility OR sterility OR fertility OR subfertility)

AND TITLE-ABS-KEY(vaginitis OR genital OR vagin*)

AND TITLE-ABS-KEY(microbiot* OR microfilm* OR microbiom* OR flora OR microflor* OR dysbiosis)

Cochrane

#1 MeSH descriptor: [Infertility] explode all trees

#2 infertility OR sterility OR fertility OR subfertility

#3 #1 OR #2

#4 MeSH descriptor: [Vaginitis] explode all trees

#5 vaginitis OR genital OR vagin*

#6 #4 OR #5

#7 MeSH descriptor: [Microbiota] explode all trees

#8 microbiot* OR microfilm* OR microbiom* OR flora OR microflor* OR dysbiosis

#9 #7 OR #8

#10 #3 AND #6 AND #9

Supplementary Material S2. Characteristics of Included Studies

Table S1. Characteristics of Included Studies

Year	First Author	16S amplified region	Primer	Primer sequence details	Details of the infertility group	Details of the control group
1997	Morgan DJ	-	-	-	Women attending a specialist infertility clinic for the first time	Women booking at an antenatal clinic.
2009	Mania-Pramanik J	-	-	-	Women attending the Obstetric and Gynaecology Outpatient Department who had not conceived within two years of marriage despite trying to conceive were classified as infertile.	The control group comprised asymptomatic non-pregnant women attending the Obstetric and Gynaecology Outpatient Department for family planning advice.
2011	Adamson PC	-	-	-	if they were married or had been with their main partner for more than two years, were sexually active, were not using modern contraception, and had no children.	women not meeting the study definition of primary infertility
2013	Salah RM	-	-	-	Women with female factor infertility, including women with unexplained infertility, which was defined as infertility for more than one year despite regular marital life, regular menstrual cycles, uneventful clinical examination, and normal husband semen analysis according to WHO criteria.	Asymptomatic fertile women attending the family planning clinic of the same hospital who agreed to participate in the study.
2013	Tomusiak A	-	-	-	Women aged treated for	Women with no history of

Year	First Author	16S amplified region	Primer	Primer sequence details	Details of the infertility group	Details of the control group
					infertility for over one year at the PARENS Centre for the Treatment of Infertility in Cracow, after exclusion of anatomical and hormonal abnormalities, endometriosis, and abnormal sperm parameters.	fertility problems and at least one child, without clinical symptoms of reproductive tract inflammation.
2015	Durugbo II	-	-	-	Women with tubal factor infertility attending follow-up at the gynecology clinic. Tubal factor infertility was previously diagnosed by hysterosalpingography. Women were excluded if they had infertility due to causes other than tubal factors or had undergone bilateral salpingectomy.	Fertile women attending the family planning clinic of the same hospital.
2017	Babu G	-	-	-	Non-pregnant women with primary or secondary infertility attending the infertility clinic at Sri Lakshmi Narayana Medical College and Hospital, Puducherry. Women with chronic autoimmune or inflammatory conditions, recent antimicrobial use, intrauterine device or hormonal contraceptive use, or symptoms of discharge, itching, burning or dysuria were	Healthy non-pregnant women with regular menstrual cycles and without any gynaecological disorder. Women with chronic autoimmune or inflammatory conditions, recent antimicrobial use, intrauterine device or hormonal contraceptive use, or symptoms of discharge, itching, burning or dysuria were excluded

Year	First Author	16S amplified region	Primer	Primer sequence details	Details of the infertility group	Details of the control group
					excluded.	
2017	Campisciano G	-	-	-	Infertile women attending the Assisted Reproductive Technology (ART) clinic of the Mother and Child Health Hospital — IRCCS Burlo Garofolo, Trieste, Italy. All women were of Caucasian origin, non-pregnant, of reproductive age, aged 32–40 years, with no current use of tobacco, alcohol, or contraceptive methods. They had no hospitalization and no use of systemic medication for chronic diseases, antibiotics, or probiotics, either oral or topical, within 6 months before sample collection. Samples were collected 5–7 days before the menstrual period and before programmed in vitro fertilization practice.	Fertile women attending the Gynecology clinic of the same Mother and Child Health Hospital — IRCCS Burlo Garofolo, Trieste, Italy. The control group included women attending for regular check-up and women attending because of vaginal complaints such as discharge, itching, burning, or dysuria. All women were of Caucasian origin, non-pregnant, of reproductive age, aged 32–40 years, with no current use of tobacco, alcohol, or contraceptive methods. They had no hospitalization and no use of systemic medication for chronic diseases, antibiotics, or probiotics, either oral or topical, within 6 months before sample collection. Samples were collected 5–7 days before the menstrual period.
2018	Wee BA	V1–V3 region	27F/534R	5' AGAGTTTGATYMTGGCTCAG3'(four oligo mix) 5'	Women with a recent previous or current history of infertility who were already undergoing hysteroscopy. Cases included women currently attending	Women with no history of infertility who were already undergoing hysteroscopy. Controls had a history of pregnancies without the need for

Year	First Author	16S amplified region	Primer	Primer sequence details	Details of the infertility group	Details of the control group
				AGGGTTCGATTCT GGCTCAG3' (Bifidobacterium-specific) 5' AGAGTTTGATCCT GGCTTAG3'(Borrelia species-specific) 5' AGAATTTGATCTT GGTTCAG3'(Chlamydia species-specific) 534R primer: 5' TTACCGCGGCTGC TGGCA3'.	gynaecological or fertility care, or women attending for other gynaecological reasons but with a recent previous history of infertility requiring assisted reproductive technologies. Inclusion criteria were women who had tried to achieve pregnancy for more than one year and who required assisted reproductive technologies to achieve pregnancy	assisted reproductive technologies and were attending gynaecological care for hysteroscopy, including hysteroscopy for intrauterine system/device change or hysterectomy, excluding suspected reproductive cancers.
2018	Kyono K	V4 hypervariable region	515F / 806RB	5'-TCGTCGGCAGC GTCAGATGTGTAT AAGAGACAGGTG YCAGCMGCCGCG GTAA-3' 5'-GTCTCGTGGGC TCGGAGATGTGTA TAAGAGACAGGG ACTACNVGGGTW TCTAAT-3'	Infertile patients, including IVF and non-IVF patients, who underwent endometrial and vaginal microbiome analysis. Participants had no symptoms suggestive of vaginitis or endometritis	Asymptomatic healthy volunteers whose endometrial and vaginal microbiomes were analyzed.
2019	Moragiamni D	-	-	-	Infertile Greek females of reproductive age, who were enrolled in an IVF program and underwent vaginal fluid examination as part of prenatal	Fertile Greek females of reproductive age, who had at least one child and underwent vaginal fluid examination as part of prenatal screening at the same

Year	First Author	16S amplified region	Primer	Primer sequence details	Details of the infertility group	Details of the control group
					screening at the Department of Microbiology, Aretaieion General Hospital, National and Kapodistrian University of Athens.	hospital during the same study period.
2020	Hasn RA	-	-	-	Women of reproductive age, who were non-pregnant and non-menstruating, had not taken any antibiotics for at least four weeks, and attended the infertility clinic with unexplained infertility.	Healthy women of reproductive age, who were non-pregnant and non-menstruating, had regular menstrual cycles, no gynecological disorders, and had not taken any antibiotics for at least four weeks.
2020	Zhao C	V1–V2 hypervariable region	27F / 355R	5'-AGAGTTTGATCMTGGCTCAG-3' (Forward primer) 5'-GCTGCCTCCCGTAGGAGT-3' (Reverse primer)	Women with secondary infertility recruited from the Affiliated Hospital of Jining Medical College. A total of 30 patients with secondary infertility were recruited and consented to participate. Inclusion criteria included age 23–42 years, not pregnant, HIV negative, no clinically significant treatments within 4 weeks before study start, regular menstrual cycles of 25–35 days, no family genetic disease, no clinically obvious inflammation, and no sexual activity within 2 weeks. Patients did not have signs or symptoms of cervical, uterine, or tubal	Healthy women recruited from the same hospital and compared with women with secondary infertility. A total of 92 healthy women were screened, including 42 women in ovulation and 50 women in the non-ovulation follicular phase. Inclusion criteria included age 23–42 years, not pregnant, HIV negative, no clinically significant treatments within 4 weeks before study start, regular menstrual cycles of 25–35 days, no family genetic disease, no clinically obvious inflammation, and no sexual activity within 2 weeks.

Year	First Author	16S amplified region	Primer	Primer sequence details	Details of the infertility group	Details of the control group
					infection.	
2021	Sun N	V1–V3 region	8F / 518R	5'-AGAGTTTGATYMTGGCTCAG-3'<br><br>5'-ATTACCGCGGCTGCTGG-3'	Infertile reproductive-aged women recruited from the Physical Examination Center of Shanghai Changzheng Hospital. General exclusion criteria included IUD use, vaginal inflammation, acute inflammation, suspected cervical or endometrial neoplasia, endocrine or autoimmune disorders, recent hormones, antibiotics, vaginal medications, recent cervical/endometrial procedures, recent douching, recent sexual activity, pregnancy, lactation, or menstruation at sampling.	Healthy reproductive-aged women recruited from the Physical Examination Center of Shanghai Changzheng Hospital. The same general exclusion and sampling criteria were applied.
2021	Azpiroz MA	V4 hypervariable region	515F / 806RB	5'-TCGTCGGCAGCGTCAGATGTGTATAAGAGACAGGTGCCAGCMGCCGCGGTAA-3'<br>5'-TCTCGTGGGCTCGGAGATGTGTATAAGAGACAGGGACTACNVGGGTWTCTAAT-3'	Women with unexplained infertility (UI) recruited. Patients were defined as UI if they met the following criteria: BMI <25; normal ovarian function, defined as cycle day 3 ± 2 days FSH ≤12 IU/L within 1 year before study initiation; normal tubal and peritoneal anatomy determined by hysterosalpingography and/or laparoscopy; midluteal serum progesterone >10 ng/mL; no	Fertile women recruited as the control group. Women in the fertile control group were aged 21–39 years, had BMI <25, and had at least one healthy baby younger than 2 years who was conceived without assisted reproductive technologies. Women were excluded from the fertile group if they were pregnant and/or breastfeeding, were under hormonal treatment,

Year	First Author	16S amplified region	Primer	Primer sequence details	Details of the infertility group	Details of the control group
					evidence of male infertility; and history of at least 2 unsuccessful IVF-ET procedures or 1 unsuccessful ovodonation procedure. Criteria related to ovarian function and tubal/peritoneal anatomy did not apply to patients in an ovodonation program because of ovarian failure diagnosis. Patients were excluded if they had hydrosalpinx, severe endometriosis, antibiotic treatment, or untreated hormonal disorders such as high prolactin level, insulin insensitivity, hypothyroidism, or hyperthyroidism.	were taking antibiotics, used an intrauterine device, or had a personal history of endocrine disease, autoimmune disease, infertility, or recurrent miscarriages.
2021	Sezer O	-	-	-	Women diagnosed with unexplained infertility and followed up in the Gynecology and Medical Genetics outpatient service between September 2019 and March 2020. Unexplained infertility was diagnosed according to the ICMART and WHO revised glossary of ART terminology after excluding common causes of infertility through standard fertility	Fertile women with a history of healthy delivery who were followed up in the Gynecology and Medical Genetics outpatient service.

Year	First Author	16S amplified region	Primer	Primer sequence details	Details of the infertility group	Details of the control group
					investigations, including semen analysis, ovulation evaluation, and tubal patency testing. A total of 26 unexplained infertility patients were included.	
2021	Lokken EM	-	-	-	Women who were non-pregnant at enrollment and had immediate fertility intent but did not become pregnant during the preconception follow-up period. Participants were from the Microbiota and Preterm Birth Study in Nairobi and Mombasa, Kenya. Eligible women were planning to conceive, HIV-negative, aged ≤45 years, and had a menstrual period in the prior 3 months or had recently discontinued an implant, hormonal IUD, or DMPA. Women with a history of infertility care-seeking or potential subfertility-related conditions were excluded. Therefore, this group represents women who remained non-pregnant during follow-up rather than women clinically diagnosed with infertility.	Women who were non-pregnant at enrollment, had immediate fertility intent, and became pregnant during the preconception follow-up period. These women were also enrolled in the Microbiota and Preterm Birth Study in Nairobi and Mombasa, Kenya, and met the same eligibility criteria, including planning to conceive, HIV-negative status, age ≤45 years, and recent menstruation or recent discontinuation of hormonal contraception.
2021	Ichiyama T	V4	515f /	5’-TCGTCGGCAGC	Women diagnosed with recurrent	Healthy women whose infertility

Year	First Author	16S amplified region	Primer	Primer sequence details	Details of the infertility group	Details of the control group
		hypervariable region	806rB	GTCAGATGTGTATAAGAGACAGGTGYCAGCMGCCGCGGTAA-3’ 5’-GTCTCGTGGGCTCGGAGATGTGTATAAGAGACAGGGACTACNVGGGTWTCTAAT-3’	implantation failure (RIF). RIF was defined as failure to achieve clinical pregnancy after at least three embryo transfer cycles using blastocysts graded 3BB or higher according to the Gardner scoring system, 5–6 days after fertilization. Clinical pregnancy was defined as the observation of a fetal sac in the uterus by transvaginal ultrasound.	etiology was male factor infertility due to their husbands’ azoospermia. These women had regular menstrual cycles and no identifiable female causes of infertility, including tubal factors, ovulation disorder, endometriosis, endocrinologic abnormalities, or immunological abnormalities.
2021	Chen H	V3–V4 hypervariable regions	341F / 806R	5′-CCTAYGGGRBGCASCAG-3′ 5′-GGACTACNNGGGTATCTAAT-3′	Women with tubal infertility who sought assistance at the assisted reproductive technology center of a teaching hospital in Chenzhou. They are trachomatis-negative tubal infertile women	Women who were C. trachomatis-negative and attended the physical examination center of the same teaching hospital in Chenzhou for an annual physical examination during the same study period. These women were classified as the control group.
2021	Manzoor A	V4 region	NA	NA	Infertile females recruited through contacts with local clinics and universities. Initially, couples who had been actively trying to achieve pregnancy for more than 1 year without conception were categorized as infertile. Exclusion criteria included antibiotic use or travel abroad within the previous 3	Fertile females recruited through contacts with local clinics and universities. Exclusion criteria included antibiotic use or travel abroad within the previous 3 months, menopause, and inflammatory bowel disease.

Year	First Author	16S amplified region	Primer	Primer sequence details	Details of the infertility group	Details of the control group
					months, menopause, and inflammatory bowel disease.	
2022	Patel N	V2–V3 hypervariable region	101F / 518R	5'-ACTGGCGGACGGGTGAGTAA-3' 5'-CGTATTACCGCGGCTGCTGG-3'	Infertile women referred to Akanksha Hospital and Research Institute. The infertile women were divided into RIF group and UE group. The RIF group included women who could not conceive after ≥2 fresh IVF-embryo transfer cycles/ICSI, or had ≥3 consecutive miscarriages. The UE group included women with unexplained infertility, diagnosed when the cause remained undefined after routine fertility tests, with infertility for more than 1 year, normospermic male partner, normal menstrual rhythm with regular ovulation, bilateral tubal patency verified by hysterosalpingogram or laparoscopy, and normal hormonal tests including thyroid, prolactin, and AMH. Exclusion criteria included diabetes, polycystic ovary syndrome, endometriosis, diarrhoea, ongoing pregnancy, addiction such as drugs, alcohol, or	Fertile women referred to Akanksha Hospital and Research Institute and assigned to the control group. The same exclusion criteria were applied, including diabetes, polycystic ovary syndrome, endometriosis, diarrhoea, ongoing pregnancy, addiction such as drugs, alcohol, or tobacco, and antibiotic use within at least 2 weeks before sample collection.

Year	First Author	16S amplified region	Primer	Primer sequence details	Details of the infertility group	Details of the control group
					tobacco, and antibiotic use within at least 2 weeks before sample collection.	
2022	Wang L	-	-	-	Infertile women diagnosed at the Reproductive Medicine Centre of Chengdu Women's and Children's Central Hospital, School of Medicine, University of Electronic Science and Technology of China. Infertility was defined as failure to conceive after at least 1 year of regular sexual intercourse without using any contraceptive measures. Participants had not used hormones, antibiotics, or immunosuppressive agents in the previous 2 weeks, had no sexual intercourse, and had no history of vulvar/vaginal medication use in the previous 3 days before sampling.	Healthy women of childbearing age recruited from the Department of Gynaecology during the same period. Inclusion criteria for controls were being married, regular menstruation, and natural pregnancy.
2022	Sethi S	-	-	-	Female patients who presented to the Regional STI Reference, Research and Training Laboratory, Department of Medical Microbiology, PGIMER, Chandigarh, and were classified as having infertility, including primary infertility or	Female patients from the same study population who did not have primary or secondary infertility. These included women presenting with symptoms of RTI/STI at the tertiary care hospital, high-risk groups such as female sex

Year	First Author	16S amplified region	Primer	Primer sequence details	Details of the infertility group	Details of the control group
					secondary infertility. Patients were not currently receiving treatment for RTI/STI. Pregnant women, HIV-positive, HBsAg-positive, hepatitis C virus-positive women, and women from whom only blood specimens were received were excluded.	workers, and women from the slum population visiting the dispensary attached to the institute. Patients were not currently receiving treatment for RTI/STI. Pregnant women, HIV-positive, HBsAg-positive, hepatitis C virus-positive women, and women from whom only blood specimens were received were excluded.
2022	Hong X	-	-	-	Women/couples from the Free Pre-pregnancy Health Examination Project at the Maternal and Child Center of Gulou District, Nanjing, China,, who were ready to become pregnant but did not achieve pregnancy within 12 months of follow-up. Women were older than 20 years, male partners were older than 22 years, and both partners were younger than 49 years. Women who were already pregnant at enrollment, had fertility-related diseases, refused vaginal swab collection, had used antibiotics within the previous 2 weeks, or were lost to follow-up were excluded.	Women/couples from the same pre-pregnancy cohort who were ready to become pregnant and achieved pregnancy within 12 months of follow-up. Women were older than 20 years, male partners were older than 22 years, and both partners were younger than 49 years. The same exclusion criteria were applied, including pregnancy at enrollment, medical conditions unsuitable for pregnancy, fertility-related diseases, refusal to provide vaginal swabs, antibiotic use within the previous 2 weeks, and loss to follow-up.

Year	First Author	16S amplified region	Primer	Primer sequence details	Details of the infertility group	Details of the control group
2023	Sethi S	-	-	-	Females who presented with infertility as one of the chief complaints and were enrolled at the Regional STIs Reference, Research and Training Center, Postgraduate Institute of Medical Education and Research, Chandigarh. Two vaginal swabs were collected during routine pelvic examination by trained healthcare professionals using FLOQSwabs™. Women were excluded if they had received antibiotics or antifungals in the preceding week, were menstruating or pregnant, or had genital tuberculosis or malignancies.	Females who presented with chief complaints other than infertility, such as vaginal discharge, burning micturition, itching, or lower abdominal pain, during the same study period and at the same center. Two vaginal swabs were collected during routine pelvic examination by trained healthcare professionals using FLOQSwabs™. The same exclusion criteria were applied, including recent antibiotic or antifungal use, menstruation, pregnancy, genital tuberculosis, or malignancies.
2023	Dong YH	-	-	-	Infertile female patients undergoing IVF treatment at the Department of Reproductive Medicine, The First People's Hospital of Yunnan Province, China. All samples were collected within 1 month to reduce the influence of climate and hospital-environment variation. Inclusion criteria were age 20–40 years, no antibiotic or antimicrobial treatment within	Healthy control women of childbearing age who were asymptomatic and attended routine well-woman visits or preconception counselling at the same hospital. All controls had a normal pregnancy history. Inclusion criteria were age 20–40 years, no antibiotic or antimicrobial treatment within the previous 3 months, no hormonal contraception use, and

Year	First Author	16S amplified region	Primer	Primer sequence details	Details of the infertility group	Details of the control group
					the previous 3 months, no hormonal contraception use, and no sexually transmitted infection or urinary tract infection. Women were excluded if infertility was due to male factor, or if they had autoimmune diseases or endocrine diseases.	no sexually transmitted infection or urinary tract infection. Women with clinical signs or symptoms suggestive of infertility, including dysmenorrhea, dyspareunia, menstrual disorder, and/or amenorrhea, were excluded from the control group.
2023	Bar O	NA	NA	NA	Women with unexplained infertility undergoing IVF.	Women undergoing IVF for male factor infertility, used as the control group.
2024	Van den Tweel M	-	-	-	Persons above 18 years old who were included after informed consent at the initial fertility assessment at Haaglanden Medical Center, The Hague, the Netherlands. Participants were excluded if they had a history of three or more miscarriages, could not understand Dutch or English, or used prophylactic antibiotic treatment. Vaginal swabs and vaginal pH measurements were collected at baseline, and participants were followed for fertility treatment and pregnancy outcomes. Follow-up ended when participants no longer wished to conceive, had a live	Persons above 18 years old who were included after informed consent at the initial fertility assessment at Haaglanden Medical Center, The Hague, the Netherlands. Participants were excluded if they had a history of three or more miscarriages, could not understand Dutch or English, or used prophylactic antibiotic treatment. Vaginal swabs and vaginal pH measurements were collected at baseline, and participants were followed for fertility treatment and pregnancy outcomes. Follow-up ended when participants no longer wished to conceive, had a live

Year	First Author	16S amplified region	Primer	Primer sequence details	Details of the infertility group	Details of the control group
					birth, ended the relationship, reached age 43 years, or reached the end of the study in October 2022.This group represents women who remained non-pregnant during follow-up .	birth, ended the relationship, reached age 43 years, or reached the end of the study in October 2022.This group represents women who remained pregnant during follow-up .
2024	Chopra C	V3–V4 hypervariable region	NA	NA	Women with idiopathic infertility recruited from the Department of Obstetrics and Gynaecology, Governmental Medical College Jammu, and its affiliated hospitals in Jammu and Kashmir, India. Exclusion criteria included history of pre-term delivery, uterine and fallopian tube malformations, vaginal infections with symptoms such as unusual vaginal discharge, genital itching and burning, abdominal pain, yeast infections, ovarian cysts, urinary tract infections, menstrual irregularity, and recent or prolonged antibiotic use.	Healthy women recruited from the same Department of Obstetrics and Gynaecology, Governmental Medical College Jammu, and affiliated hospitals during the same period. The control cohort included 40 healthy women who had a previous full-term delivery. The same exclusion criteria were applied, including history of pre-term delivery, uterine and fallopian tube malformations, vaginal infections, yeast infections, ovarian cysts, urinary tract infections, menstrual irregularity, and recent or prolonged antibiotic use.
2025	Chen X	V3–V4 hypervariable region	338F / 806R	5'-ACTCCTACGGGAGGCAGCAG-3' 5'-GACTACHVGGGTWTCTAAT-3'	Women diagnosed with infertility at the Reproductive Medicine Center of Shanghai Changzheng Hospital, China. A total of 194 infertile women were enrolled as the infertile group.	Healthy women who underwent routine physical examinations at the Physical Examination Center of Shanghai Changzheng Hospital during the same study period. The inclusion criterion

Year	First Author	16S amplified region	Primer	Primer sequence details	Details of the infertility group	Details of the control group
					The inclusion criterion was regular unprotected sexual activity for at least 1 year without achieving pregnancy. Exclusion criteria included presence of an intrauterine device, vaginal inflammation, any acute inflammatory condition, suspected cervical or endometrial neoplasia, and endocrine or autoimmune disorders. Women were also excluded if they had recently used hormones, antibiotics, or vaginal medications; undergone cervical treatment, endometrial biopsy, IUD removal, or hysteroscopy within the previous week; engaged in douching within 5 days; or had sexual activity within 48 hours before sampling. None of the participants were pregnant, lactating, or menstruating at the time of sampling.	was a history of childbirth with no record of infertility or other gynecological diseases. The same exclusion criteria were applied: presence of an intrauterine device, vaginal inflammation, any acute inflammatory condition, suspected cervical or endometrial neoplasia, endocrine or autoimmune disorders, recent use of hormones, antibiotics, or vaginal medications, cervical treatment, endometrial biopsy, IUD removal, or hysteroscopy within the previous week, douching within 5 days, or sexual activity within 48 hours before sampling. None were pregnant, lactating, or menstruating at the time of sampling.
2025	Zhao H	V3 – V4 region	338F / 806R	5'-ACTCCTACGGGAGGCAGCA-3' 5'-GGACTACHVGGGTWTCTAAT-3'	Infertile women recruited from the Reproductive Center of Guiyang Maternal and Child Health Hospital in China. Infertility was clinically	Fertile women recruited from the same hospital during the same period who underwent ovulation monitoring, assigned to the control group. Inclusion criteria

Year	First Author	16S amplified region	Primer	Primer sequence details	Details of the infertility group	Details of the control group
					diagnosed as failure to conceive after at least 1 year of normal sexual life without contraception. PCOS infertility participants met both the diagnostic criteria for PCOS and infertility. TFI participants had infertility for 12 months without contraception and tubal abnormalities confirmed by tubography, including interstitial tubal obstruction, hydrosalpinx, or incomplete patency; they were married and had a normal sexual history. Women with genital system malformations or organic diseases, infertility caused by factors other than ovulation disorders or TFI, or abnormal semen examination in the male partner were excluded. Participants with mental illness, systemic immune diseases, major somatic diseases such as malignant tumors, positive TCT or TORCH/HIV results were also excluded.	were no history of PCOS or other endocrine diseases, confirmed tubal patency based on tubography, transvaginal ultrasound examination combined with laboratory examination of sex hormones, and normal ovarian reserve function. Participants in the group should not have taken hormonal medications or antibiotics, or undergone other vaginal preparations within 1 month, to avoid disruption of the vaginal flora. Participants with mental illness, systemic immune diseases, major somatic diseases such as malignant tumors, positive TCT or TORCH/HIV results were excluded.

Supplementary Material S3. Quality Assessment

Table S2. Quality Assessment of Cross-Sectional Studies (AHRQ Scale)

Year	First Author	Study Design	1	2	3	4	5	6	7	8	9	10	11	Total	Quality
1997	Morgan DJ	Cross-section	Yes	No	No	No	Yes	Unclear	No	No	No	No	Yes	3	Low
2009	Mania-Pramanik J	Cross-section	Yes	No	No	No	Yes	Unclear	Yes	No	Yes	No	Yes	5	Medium
2013	Salah RM	Cross-section	Yes	Yes	Yes	Yes	Yes	Unclear	Yes	Yes	Yes	Yes	Yes	10	High
2013	Tomusiak A	Cross-section	Yes	Yes	No	No	Yes	Unclear	Yes	No	Unclear	No	Yes	5	Medium
2015	Durugbo II	Cross-section	Yes	Yes	Yes	Yes	Yes	Unclear	Yes	No	No	Yes	Yes	8	Medium
2017	Babu G	Cross-section	Yes	Yes	Yes	No	Yes	Unclear	Yes	No	Yes	No	Yes	7	Medium
2017	Campisciano G	Cross-section	Yes	Yes	Yes	No	Yes	Yes	Yes	No	No	No	Yes	7	Medium
2018	Wee BA	Cross-section	Yes	Yes	No	No	Yes	Yes	Yes	No	Yes	No	Yes	7	Medium
2018	Kyono K	Cross-section	Yes	Yes	Yes	No	Yes	Yes	No	No	No	No	Yes	6	Medium
2019	Moragiamni D	Cross-section	Yes	No	Yes	No	Yes	Unclear	Yes	No	Yes	No	Yes	6	Medium
2020	Hasn RA	Cross-section	Yes	Yes	Yes	No	Yes	Yes	Yes	No	Yes	No	Yes	8	Medium
2020	Zhao C	Cross-section	Yes	Yes	Yes	No	Yes	Yes	Yes	No	Yes	No	Yes	8	Medium
2021	Sun N	Cross-section	Yes	Yes	No	No	Yes	Yes	Yes	No	No	No	Yes	6	Medium
2021	Azpiroz MA	Cross-section	Yes	Yes	Yes	No	Yes	Yes	Yes	No	No	No	Yes	7	Medium
2021	Sezer O	Cross-section	Yes	Yes	Yes	No	Yes	Yes	Yes	Yes	Yes	No	Yes	9	High
2021	Ichiyama T	Cross-section	Yes	Yes	Yes	Yes	Yes	Yes	Yes	No	Yes	No	Yes	9	High
2021	Chen H	Cross-section	Yes	Yes	Yes	No	Yes	Yes	Yes	No	Yes	No	Yes	9	High

Year	First Author	Study Design	1	2	3	4	5	6	7	8	9	10	11	Total	Quality
2021	Manzoor A	Cross-section	Yes	Yes	No	No	Yes	Yes	Yes	No	No	No	Yes	6	Medium
2022	Patel N	Cross-section	Yes	Yes	Yes	No	Yes	Yes	No	No	No	No	Yes	6	Medium
2022	Wang L	Cross-section	Yes	Yes	Yes	No	Yes	Yes	No	No	No	No	Yes	6	Medium
2022	Sethi S	Cross-section	Yes	Yes	Yes	No	Yes	Yes	Yes	No	No	No	Yes	7	Medium
2023	Sethi S	Cross-section	Yes	Yes	Yes	No	Yes	No	Yes	No	Yes	No	Yes	7	Medium
2023	Dong YH	Cross-section	Yes	Yes	Yes	No	Yes	Yes	Yes	No	Yes	No	Yes	8	Medium
2023	Bar O	Cross-section	Yes	No	No	No	Yes	Unclear	No	No	No	No	Yes	3	Low
2025	Chen X	Cross-section	Yes	Yes	Yes	No	Yes	Yes	No	Yes	Yes	No	Yes	8	Medium

Table S3. Quality Assessment of Analytical Studies (NOS Scale)

Year	First Author	Study Design	1	2	3	4	1	1	2	3	Total	**Quality**
2011	Adamson PC	Nest Case-control	0	1	0	1	0	1	1	0	4	Low
2025	Zhao H	Case-control	1	0	0	1	0	1	1	0	4	Low
2021	Lokken EM	Prospective Cohort	1	1	1	1	1	1	1	1	8	High
2022	Hong X	Prospective Cohort	1	1	1	1	0	1	1	0	6	Low
2024	Van den Tweel M	Prospective Cohort	1	1	1	1	1	1	1	1	8	High
2024	Chopra C	Prospective Cohort	1	1	1	1	0	1	1	1	6	Low

Supplementary Material S4. Sensitivity analysis

Table S4. Meta-Regression Analysis of the Association Between LL-VMB and Infertility

Predictor	Estimate	SE	z	*p*	95% CI (Lower)	95% CI (Upper)
intrcpt	-0.3219	2.2961	-0.1402	0.8885	-4.8223	4.1784
Country: India	0.2634	1.4404	0.1829	0.8549	-2.5597	3.0865
Country: Others[a]	-0.3137	0.5986	-0.5240	0.6003	-1.4869	0.8596
Study type: Prospective Cohort	-1.8006	1.5681	-1.1483	0.2509	-4.8740	1.2728
Study type: Others[b]	0.5795	1.5828	0.3661	0.7143	-2.5227	3.6817
Type of infertility: Others[c]	0.2887	0.7091	0.4071	0.6839	-1.1012	1.6786
Type of control women: Healthy non-pregnant women (no history of infertility, pregnancy history unspecified)	-0.5927	0.6446	-0.9195	0.3578	-1.8561	0.6707
Type of control women: Others[d]	1.4446	2.2872	0.6316	0.5277	-3.0382	5.9274
Diagnosis method: Sequencing	0.4631	1.4776	0.3134	0.7539	-2.4329	3.3592
Diagnosis method: Others[e]	0.8073	1.2312	0.6557	0.5120	-1.6058	3.2204
Quality: High	1.6190	1.1754	1.3774	0.1684	-0.6847	3.9226
Quality: Middle	1.2679	1.2552	1.0101	0.3124	-1.1923	3.7282

Notes: [a]Countries include: Egypt, Poland, Nigeria, Australia, Greece, Iraq, Turkey, Holland, Japan, Kenyan, Australia, Turkey

[b]Study types include: Nest case-control, case-control

[c]Infertility types include: Primary infertility, Secondary infertility, Tubal factor infertility, Ovulatory disorders, Uterine factors, Unexplained infertility, Natural conception cohorts: participants who failed to conceive after follow-up.

[d]Infertility types include: Antenatal women, Healthy non-pregnant women with a history of pregnancy

[e]Diagnostic methods include: Spiegel, PCR, Amsel, Nugent+Amsel

Table S5. Meta-Regression Analysis of the Association Between BV and Infertility

Predictor	Estimate	SE	z	*p*	95% CI (Lower)	95% CI (Upper)
intrcpt	4.5155	3.8901	1.1608	0.2457	-3.1089	12.1400

Predictor	Estimate	SE	z	*p*	95% CI (Lower)	95% CI (Upper)
Country: India	-1.6182	1.9647	-0.8237	0.4101	-5.4690	2.2325
Country: Others[a]	-0.3333	0.8396	-0.3970	0.6914	-1.9790	1.3123
Study type: Prospective Cohort	-0.2331	2.1574	-0.1080	0.9140	-4.4616	3.9954
Study type: Others[b]	-3.0643	2.6720	-1.1468	0.2515	-8.3014	2.1728
Type of infertility: Others[c]	0.7572	0.8101	0.9347	0.3499	-0.8306	2.3450
Type of control women: Healthy non-pregnant women (no history of infertility, pregnancy history unspecified)	-0.3442	0.8609	-0.3999	0.6892	-2.0315	1.3430
Type of control women: Others[d]	-3.2806	3.7354	-0.8782	0.3798	-10.6019	4.0407
Diagnosis method: Sequencing	-0.6614	2.2513	-0.2938	0.7689	-5.0738	3.7511
Diagnosis method: Others[e]	-1.3675	1.7882	-0.7647	0.4444	-4.8723	2.1374
Quality: High	-0.8892	2.1007	-0.4233	0.6721	-5.0066	3.2281
Quality: Middle	-1.9201	2.3378	-0.8213	0.4115	-6.5022	2.6619

Notes: [a]Countries include: Egypt, Poland, Nigeria, Australia, Greece, Iraq, Turkey, Holland, Japan, Kenyan, Australia, Turkey

[b]Study types include: Nest case-control, case-control

[c]Infertility types include: Primary infertility, Secondary infertility, Tubal factor infertility, Ovulatory disorders, Uterine factors, Unexplained infertility, Natural conception cohorts: participants who failed to conceive after follow-up.

[d]Infertility types include: Antenatal women, Healthy non-pregnant women with a history of pregnancy

[e]Diagnostic methods include: Spiegel, PCR, Amsel, Nugent+Amsel

Table S6. Subgroup analysis of the Association Between LL-VMB and Infertility

Subgroup	Articles	OR	95% CI (Lower)	95% CI (Upper)	P	I^2
Country						
India	5	1.7078	0.8829	3.3031	0.1118	81.0600
China	4	2.5376	1.9387	3.3217	<0.0001	0.0000
Others[a]	14	2.3941	1.5758	3.6373	<0.0001	68.6066
Study type						
Cross-section	18	2.4612	1.7021	3.5587	<0.0001	84.3547
Case-control	2	1.1183	0.7484	1.671	0.5852	0.0000
Prospective Cohort	3	1.4626	0.8875	2.4105	0.1358	28.4320
Type of infertility						
Primary infertility	1	1.1043	0.7365	1.6558	0.6313	-
Tubal factor infertility	1	4.5759	2.4222	8.6445	<0.0001	-
Unexplained infertility	1	3.375	0.349	32.6372	0.2934	-
Clinical population from infertility clinics or undergoing ART/IVF/ICSI	17	2.321	1.5769	3.4164	<0.0001	83.7424
Natural conception cohorts: participants who failed to conceive after follow-up	3	1.4626	0.8875	2.4105	0.1358	28.4320
Type of control						
Antenatal women	1	2.2456	1.5634	3.2255	<0.0001	-
Healthy non-pregnant women with a history of pregnancy	4	4.4267	2.0158	9.7209	0.0002	0.0000
Healthy non-pregnant women (no history of infertility, pregnancy history	15	2.0504	1.3462	3.1229	0.0008	85.5470

Subgroup	Articles	OR	95% CI (Lower)	95% CI (Upper)	P	I^2
unspecified)						
Healthy non-pregnant women who subsequently became pregnant	3	1.4626	0.8875	2.4105	0.1358	28.4320
Diagnosis method						
Amsel	2	2.3791	0.6314	8.9646	0.2003	85.0975
Nugent	9	1.8806	1.2337	2.8667	0.0033	80.8250
PCR	4	2.5957	0.9608	7.0126	0.0600	72.8953
Sequencing	6	1.5962	0.6469	3.9382	0.3102	33.3302
Spiegel	2	3.5417	1.7474	7.1784	0.0005	84.1054
Quality						
Low	6	1.4233	0.9148	2.2144	0.1175	63.7267
Middle	12	2.3589	1.5316	3.633	<0.0001	84.1157
High	5	3.1787	1.5552	6.497	0.0015	61.4971

Notes: [a]Countries include: the UK, Egypt, Poland, Nigeria, Australia, Italy, Japan, Greece, Iraq, the USA, Turkey, Kenya, the Netherlands, and Pakistan.

Table S7. Subgroup analysis of the Association Between BV and Infertility

**Subgroup**	**Articles**	**OR**	**95% CI (Lower)**	**95% CI (Upper)**	**P**	I^2
Country						
China	3	2.5443	1.9182	3.3747	<0.0001	0.0000
India	5	1.7270	0.8888	3.3558	0.1070	80.4457
Others[a]	9	2.4953	1.5879	3.9212	<0.0001	72.5120
Study type						
Case-control	2	1.2072	0.7663	1.9018	0.4168	0.0000
Cross-section	13	2.5439	1.7016	3.8034	<0.0001	84.1123
Prospective Cohort	2	1.2132	0.7923	1.8577	0.3740	0.0000
Type of infertility						
Primary infertility	1	1.1898	0.7514	1.8839	0.4587	-
Tubal factor infertility	1	4.5759	2.4222	8.6445	<0.0001	-
Unexplained infertility	1	3.7692	0.1773	80.1225	0.3949	-
Clinical population from infertility clinics or undergoing ART/IVF/ICSI	12	2.3755	1.5610	3.6150	<0.0001	83.8527
Natural conception cohorts: participants who failed to conceive after follow-up	2	1.2132	0.7923	1.8577	0.3740	0.0000
Type of control						
Antenatal women	1	2.4635	1.6444	3.6908	<0.0001	-
Healthy non-pregnant women with a history of pregnancy	3	2.9928	1.6260	5.5086	0.0004	0.0000
Healthy non-pregnant women (no history of infertility, pregnancy history unspecified)	11	2.2665	1.4182	3.6224	0.0006	86.5282

Subgroup	Articles	OR	95% CI (Lower)	95% CI (Upper)	P	I^2
Healthy non-pregnant women who subsequently became pregnant	2	1.2132	0.7923	1.8577	0.3740	0.0000
Diagnosis method						
Amsel	2	2.3791	0.6314	8.9646	0.2003	85.0975
Nugent	8	1.8495	1.1589	2.9517	0.0099	79.0967
Nugent+Amsel	1	2.7984	1.4790	5.2949	0.0016	-
Nugent+Sequencing	1	6.9665	0.3997	121.4294	0.1832	-
PCR	2	1.0960	0.6673	1.8004	0.7172	0.0000
Sequencing	1	2.3006	0.1078	49.1104	0.5937	-
Spiegel	2	3.5417	1.7474	7.1784	0.0005	84.1054
Quality						
High	3	3.4038	1.3412	8.6380	0.0099	64.3320
Middle	10	2.2833	1.4317	3.6414	0.0005	83.5712
Low	4	1.5001	0.9010	2.4975	0.1189	65.0018

Notes: [a]Countries include: the UK, Egypt, Poland, Nigeria, Australia, Italy, Japan, Greece, Iraq, the USA, Turkey, Kenya, the Netherlands, and Pakistan.

Supplementary Material S5. GRADE Assessments

Table S8. GRADE Assessment for the Association Between LL-VMB and Infertility

**Quality assessment**							**No of patients**		**Effect**		**Quality**	**Importance**
**No of studies**	**Design**	**Risk of bias**	**Inconsistency**	**Indirectness**	**Imprecision**	**Other considerations**			**Relative (95% CI)**	**Absolute**		
**Infertility**												
23	observational studies	serious[1]	serious[2]	serious[3]	no serious imprecision	none	-	-	OR 2.16 (1.59 to 2.95)	-	VERY LOW	

[1] The body of evidence was mainly derived from cross-sectional studies, with a limited number of cohort studies, and no randomized controlled trials were available. Most included studies did not adequately control for potential confounding factors.

[2] Substantial heterogeneity was observed across studies due to differences in study design and population characteristics (I^2 = 81.0%).

[3] In several studies, the relative abundance of Lactobacillus was presented only as graphical distributions, without explicit numerical reporting. Quantitative data were therefore estimated by visual extraction from figures, which may have introduced additional uncertainty.

Table S9. GRADE Assessment for the Association Between BV and Infertility

**Quality assessment**							**No of patients**		**Effect**		**Quality**	**Importance**
**No of studies**	**Design**	**Risk of bias**	**Inconsistency**	**Indirectness**	**Imprecision**	**Other considerations**			**Relative (95% CI)**	**Absolute**		

Infertility												
17	observational studies	serious[1]	serious[2]	no serious indirectness	no serious imprecision	none	-	-	OR 2.17 (1.54 to 3.06)	-	VERY LOW	

[1] The body of evidence was mainly derived from cross-sectional studies, with a limited number of cohort studies, and no randomized controlled trials were available. Most included studies did not adequately control for potential confounding factors.

[2] Substantial heterogeneity was observed across studies due to differences in study design and detection methods ($I^2 = 81.0\%$).

Table S10. GRADE Assessment for the Association Between Intermediate Microbiota and Infertility

**Quality assessment**							**No of patients**		**Effect**		**Quality**	**Importance**
**No of studies**	**Design**	**Risk of bias**	**Inconsistency**	**Indirectness**	**Imprecision**	**Other considerations**			**Relative (95% CI)**	**Absolute**		
**Infertility**												
3	observational studies	serious[1]	no serious inconsistency	no serious indirectness	no serious imprecision	none	-	-	OR 1.01 (0.82 to 1.23)	-	VERY LOW	

[1] The body of evidence was derived from cross-sectional studies.

Table S11. GRADE Assessment for the Association Between CSTs and Infertility

Comparison	Number of studies	Within-study bias	Reporting bias	Indirectness	Imprecision	Heterogeneity	Incoherence	Confidence rating
CST I:CST II	4	Major concerns	Some concerns	No concerns	Major concerns	No concerns	No concerns	Very Low
CST I:CST III	4	Major concerns	Some concerns	No concerns	Major concerns	No concerns	No concerns	Very Low
CST I:CST IV	4	Major concerns	Some concerns	No concerns	Major concerns	No concerns	No concerns	Very Low
CST I:CST V	4	Major concerns	Some concerns	No concerns	Major concerns	No concerns	No concerns	Very Low
CST II:CST III	4	Major concerns	Some concerns	No concerns	Major concerns	No concerns	No concerns	Very Low
CST II:CST IV	4	Major concerns	Some concerns	No concerns	Major concerns	No concerns	No concerns	Very Low
CST II:CST V	4	Major concerns	Some concerns	No concerns	Major concerns	No concerns	No concerns	Very Low
CST III:CST IV	4	Major concerns	Some concerns	No concerns	Major concerns	No concerns	No concerns	Very Low
CST III:CST V	4	Major concerns	Some concerns	No concerns	Major concerns	No concerns	No concerns	Very Low
CST IV:CST V	4	Major concerns	Some concerns	No concerns	Major concerns	No concerns	No concerns	Very Low

Supplementary Material S6. PRISMA checklist [79]

Section and Topic	Item #	Checklist item	Location where item is reported
**TITLE**			
Title	1	Identify the report as a systematic review.	1
**ABSTRACT**			
Abstract	2	See the PRISMA 2020 for Abstracts checklist.	1
**INTRODUCTION**			
Rationale	3	Describe the rationale for the review in the context of existing knowledge.	1-2
Objectives	4	Provide an explicit statement of the objective(s) or question(s) the review addresses.	2
**METHODS**			
Eligibility criteria	5	Specify the inclusion and exclusion criteria for the review and how studies were grouped for the syntheses.	3
Information sources	6	Specify all databases, registers, websites, organisations, reference lists and other sources searched or consulted to identify studies. Specify the date when each source was last searched or consulted.	3
Search strategy	7	Present the full search strategies for all databases, registers and websites, including any filters and limits used.	Supplementary Material 1
Selection process	8	Specify the methods used to decide whether a study met the inclusion criteria of the review, including how many reviewers screened each record and each report retrieved, whether they worked independently, and if applicable, details of automation tools used in the process.	4
Data collection process	9	Specify the methods used to collect data from reports, including how many reviewers collected data from each report, whether they worked independently, any processes for obtaining or confirming data from study investigators, and if applicable, details of automation tools used in the process.	4
Data items	10a	List and define all outcomes for which data were sought. Specify whether all results that were compatible with each outcome domain in each study were sought (e.g. for all measures, time points, analyses), and if not, the methods used to decide which results to collect.	4
	10b	List and define all other variables for which data were sought (e.g. participant and intervention characteristics, funding sources). Describe any	4

Section and Topic	Item #	Checklist item	Location where item is reported
		assumptions made about any missing or unclear information.	
Study risk of bias assessment	11	Specify the methods used to assess risk of bias in the included studies, including details of the tool(s) used, how many reviewers assessed each study and whether they worked independently, and if applicable, details of automation tools used in the process.	4
Effect measures	12	Specify for each outcome the effect measure(s) (e.g. risk ratio, mean difference) used in the synthesis or presentation of results.	4
Synthesis methods	13a	Describe the processes used to decide which studies were eligible for each synthesis (e.g. tabulating the study intervention characteristics and comparing against the planned groups for each synthesis (item #5)).	5
	13b	Describe any methods required to prepare the data for presentation or synthesis, such as handling of missing summary statistics, or data conversions.	4
	13c	Describe any methods used to tabulate or visually display results of individual studies and syntheses.	4
	13d	Describe any methods used to synthesize results and provide a rationale for the choice(s). If meta-analysis was performed, describe the model(s), method(s) to identify the presence and extent of statistical heterogeneity, and software package(s) used.	4-5
	13e	Describe any methods used to explore possible causes of heterogeneity among study results (e.g. subgroup analysis, meta-regression).	5
	13f	Describe any sensitivity analyses conducted to assess robustness of the synthesized results.	5
Reporting bias assessment	14	Describe any methods used to assess risk of bias due to missing results in a synthesis (arising from reporting biases).	5
Certainty assessment	15	Describe any methods used to assess certainty (or confidence) in the body of evidence for an outcome.	4
**RESULTS**			
Study selection	16a	Describe the results of the search and selection process, from the number of records identified in the search to the number of studies included in the review, ideally using a flow diagram.	5-6
	16b	Cite studies that might appear to meet the inclusion criteria, but which were excluded, and explain why they were excluded.	5
Study	17	Cite each included study and present its characteristics.	7-8 and Tables

Section and Topic	Item #	Checklist item	Location where item is reported
characteristics			S2-1
Risk of bias in studies	18	Present assessments of risk of bias for each included study.	Supplementary Material 3
Results of individual studies	19	For all outcomes, present, for each study: (a) summary statistics for each group (where appropriate) and (b) an effect estimate and its precision (e.g. confidence/credible interval), ideally using structured tables or plots.	9-13
Results of syntheses	20a	For each synthesis, briefly summarise the characteristics and risk of bias among contributing studies.	9-11
	20b	Present results of all statistical syntheses conducted. If meta-analysis was done, present for each the summary estimate and its precision (e.g. confidence/credible interval) and measures of statistical heterogeneity. If comparing groups, describe the direction of the effect.	9-11
	20c	Present results of all investigations of possible causes of heterogeneity among study results.	13
	20d	Present results of all sensitivity analyses conducted to assess the robustness of the synthesized results.	13
Reporting biases	21	Present assessments of risk of bias due to missing results (arising from reporting biases) for each synthesis assessed.	13-14
Certainty of evidence	22	Present assessments of certainty (or confidence) in the body of evidence for each outcome assessed.	9-13 and Supplementary Material 5
**DISCUSSION**			
Discussion	23a	Provide a general interpretation of the results in the context of other evidence.	14-16
	23b	Discuss any limitations of the evidence included in the review.	16
	23c	Discuss any limitations of the review processes used.	16
	23d	Discuss implications of the results for practice, policy, and future research.	16-17
**OTHER INFORMATION**			
Registration and protocol	24a	Provide registration information for the review, including register name and registration number, or state that the review was not registered.	3
	24b	Indicate where the review protocol can be accessed, or state that a protocol was not prepared.	3

Section and Topic	Item #	Checklist item	Location where item is reported
	24c	Describe and explain any amendments to information provided at registration or in the protocol.	No modification
Support	25	Describe sources of financial or non-financial support for the review, and the role of the funders or sponsors in the review.	17
Competing interests	26	Declare any competing interests of review authors.	17
Availability of data, code and other materials	27	Report which of the following are publicly available and where they can be found: template data collection forms; data extracted from included studies; data used for all analyses; analytic code; any other materials used in the review.	17

From: Page MJ, McKenzie JE, Bossuyt PM, Boutron I, Hoffmann TC, Mulrow CD, et al. The PRISMA 2020 statement: an updated guideline for reporting systematic reviews. BMJ 2021;372:n71. doi: 10.1136/bmj.n71. This work is licensed under CC BY 4.0. To view a copy of this license, visit https://creativecommons.org/licenses/by/4.0/
